# Twelve Chinese patent medicines combined with conventional medicine for the treatment of functional dyspepsia: a network meta-analysis

**DOI:** 10.3389/fmed.2025.1670153

**Published:** 2025-12-12

**Authors:** Yuanfeng Han, Wenrui Huang, Wenjiang Wu, Shaoyang Cui, Li Zhang, Lizhen Ye, Jie Lin, Nanbu Wang

**Affiliations:** 1 Shenzhen Hospital (Fu Tian) of Guangzhou University of Chinese Medicine, Shenzhen, China; 2 Shenzhen Traditional Chinese Medicine Hospital, Shenzhen, China; 3 National Key Laboratory of Syndromes of Traditional Chinese Medicine, The First Affiliated Hospital of Guangzhou University of Chinese Medicine, Guangzhou, China

**Keywords:** functional dyspepsia, Chinese patent medicines, network meta-analysis, efficacy, systematic review

## Abstract

**Background:**

This study systematically evaluates the clinical efficacy and safety of 12 Chinese Patent Medicines (CPMs) combined with conventional Western medicine (CWM) in treating Functional Dyspepsia (FD), aiming to provide robust evidence for optimizing treatment strategies.

**Methods:**

We systematically searched eight Chinese and international databases up to September 2025, including China National Knowledge Infrastructure, Wanfang Data, VIP Information, China Biology Medicine Disc, PubMed, Embase, Web of Science, and the Cochrane Library. Risk of bias was assessed using the Cochrane tool, and evidence certainty was appraised with CINeMA. Network meta-analysis was performed within a frequentist framework using random-effects models, and treatments were ranked by the surface under the cumulative ranking curve (SUCRA).

**Results:**

A total of 76 RCTs involving 7,575 participants were included. All CPM + CWM regimens were more effective than CWM alone. The most significant benefit for total effective rate was observed with Jinghua Weikang Capsules (JWC) + CWM (RR = 1.46, 95% CI 1.28–1.67; SUCRA = 98.4%), Wuling Capsules (WLC) + CWM (RR = 1.29, 95% CI 1.18–1.42; SUCRA = 80.9%), and Qizhi Weitong Granules (QWG) + CWM (RR = 1.26, 95% CI 1.14–1.38; SUCRA = 69.2%). For motilin, WLC + CWM, Zhizhu Kuanzhong Capsules (ZKC) + CWM, and Simo Tang Oral Liquid (STOL) + CWM showed significant improvements. For gastrin, Liuwei Nengxiao Capsules (LNC) + CWM, Dalitong Granules (DLTG) + CWM, and STOL + CWM ranked highest. No CPM + CWM regimen was associated with a significant increase in adverse events, except for a higher risk with WLC + CWM versus QWG + CWM (OR = 4.82, 95% CI 1.02–22.87). Sensitivity analyses and meta-regression supported the robustness of these findings, while CINeMA rated the certainty of evidence as low for most comparisons.

**Conclusion:**

CPM combined with CWM was more effective than CWM alone in improving symptom response and gastrointestinal hormone levels, without increasing adverse events. These findings support CPMs as promising adjuncts to standard therapy, though higher-quality RCTs are needed to confirm their role in personalized management of functional dyspepsia.

**Systematic review registration:**

https://www.crd.york.ac.uk/PROSPERO/view/ CRD42024562649.

## Introduction

Functional dyspepsia (FD) is a common functional gastrointestinal disorder defined by recurrent upper abdominal pain, burning, postprandial fullness, and early satiety, in the absence of structural disease confirmed by endoscopy (1). The Rome IV criteria distinguish two subtypes: epigastric pain syndrome, with pain or burning at least once a week, and postprandial distress syndrome, with bothersome fullness or early satiety at least three times a week (2). Prevalence estimates for FD range from 0.7% to 19.4% worldwide (3). FD frequently coexists with gastroesophageal reflux disease and irritable bowel syndrome (4, 5). Reported risk factors include psychological stress, older age, female sex, low body mass index, acute gastroenteritis, smoking, non-steroidal anti-inflammatory drug (NSAID) use, and *Helicobacter pylori* infection (6, 7). Pathophysiology is incompletely understood but likely involves altered gut sensory and motor function, immune disturbance, and disruption of brain–gut signalling (7).

First-line management focuses on *H. pylori* eradication when infection is present, using triple or quadruple therapy (1). Other pharmacological options include proton pump inhibitors, prokinetic drugs, and psychotropic agents, complemented by lifestyle advice or endoscopic procedures such as peroral endoscopic myotomy (8). Although FD is not life-threatening, it substantially reduces quality of life, productivity, and contributes to increased healthcare costs (9). Treatment efficacy remains unsatisfactory due to limited mechanistic understanding, lack of predictive biomarkers (10, 11), and frequent overlap with other gastrointestinal conditions (12). These challenges highlight the need for additional therapeutic approaches.

In China, Chinese patent medicines (CPMs) are widely used as complementary treatments for FD. Current guidelines recommend several formulations, including Zhizhu Kuanzhong Capsules (ZKC), Wuling Capsules (WLC), Qizhi Weitong Granules (QWG), Xiangsha Pingwei Granules (XPG), Dalitong Granules (DLTG), Weisu Granules (WSG), Jinghua Weikang Capsules (JWC), Weichang An Capsules (WCAC), Bilin Weitong Granules (BWC), Simo Tang Oral Liquid (STOL), Liuwei Nengxiao Capsules (LNC), and Liuwei Anxiao Capsules (LAC) (13, 14). Clinical studies and meta-analyses suggest that combining CPMs with conventional drugs may improve symptoms, enhance response rates, reduce adverse events, and improve quality of life compared with Western medicine alone (15, 16). Pharmacological evidence supports their effects on gastrointestinal motility, visceral sensitivity, psychological status, and gut microbiota regulation (17). However, heterogeneity among CPMs and individual variation in treatment response limit direct comparisons and the generation of high-level evidence.

Traditional meta-analyses are restricted to pairwise comparisons and cannot account for the wide range of treatment options. Network meta-analysis offers a way to integrate direct and indirect evidence, allowing comparative assessment across multiple interventions. We therefore conducted a systematic review and network meta-analysis of 12 CPMs for FD. We aimed to evaluate their relative efficacy and safety when combined with conventional therapy, and to provide robust evidence to inform treatment choices in clinical practice.

## Method

This study was designed and reported in line with the PRISMA 2020 guidelines and the extension for network meta-analyses (18, 19) and has been prospectively registered with PROSPERO (CRD42024562649). The PRISMA-NMA checklist is provided in Appendix 1.

### Literature search strategy

We conducted a comprehensive search of both Chinese and international databases to identify relevant studies on functional dyspepsia (FD). The databases included China National Knowledge Infrastructure (CNKI), Wanfang Data, VIP Information, China Biology Medicine Disc (CBM), PubMed, Embase, web of science and the Cochrane Library. The search was updated to September 2025 to ensure the inclusion of the most recent evidence. Search strategies were developed according to the PICOS framework, combining Medical Subject Headings (MeSH) terms with free-text keywords using Boolean operators (AND, OR). For example: ((“Dyspepsia”[Mesh] OR “functional dyspepsia”[tiab] OR “non-ulcer dyspepsia”[tiab]) AND (“Medicine, Chinese Traditional”[Mesh] OR “Chinese patent medicine”[tiab] OR “Zhizhu Kuanzhong”[tiab] OR “Wuling”[tiab]) AND (“Randomized Controlled Trial”[Publication Type] OR “randomized controlled trial”[tiab] OR “RCT”[tiab])). The complete electronic search strategies for all databases, including detailed Boolean search formulas and keywords for both English and Chinese databases, are provided in Appendix 2 (Tables S1–S5).

### Inclusion criteria

Inclusion criteria encompass the following four aspects: (1) Study Type: Randomized controlled trials (RCTs) published in Chinese or English. Population: Patients diagnosed with FD according to the Rome III or Rome IV criteria or relevant national consensus, with no restrictions on gender, age, duration of illness, nationality, or ethnicity. (2) Interventions: The control group received conventional pharmacological treatments, such as proton pump inhibitors (PPIs), prokinetic agents, or *Helicobacter pylori* eradication regimens, while the intervention group additionally received one of 12 oral Chinese patent medicines (CPMs): ZKC, WLC, QWG, XPG, DLTG, WSG, JWC, WCAC, BWC, STOL, LNC, or LAC. These 12 formulations were selected because they are recommended in current Chinese clinical guidelines for functional dyspepsia and are among the most widely prescribed CPMs in practice, with substantial clinical and pharmacological evidence supporting their effects on gastrointestinal motility, visceral sensitivity, psychological status, and gut microbiota regulation. There are no restrictions on the dosage and administration of the medications, with a treatment duration not exceeding 6 months. (3) Outcomes: (i) total effective rate (defined as the proportion of participants achieving either complete symptom resolution or significant improvement, commonly operationalized as a ≥ 50% reduction in overall FD symptom scores, in line with definitions used in the included trials); (ii) serum motilin (MTL) levels; (iii) serum gastrin (GAS) levels; and (iv) adverse event rate.

### Exclusion criteria

The exclusion criteria for this study are as follows: (1) Theses, dissertations, case reports, retrospective and prospective cohort studies, animal studies, reviews, and duplicated publications. (2) Studies with evident errors or unclear methodologies. (3) Research involving participants with comorbid conditions.

### Study selection and data extraction

Two researchers independently screened the literature and extracted data using a standardized form. Duplicate records were first removed, followed by exclusion of non-randomized trials or animal studies based on titles and abstracts. Full texts were then reviewed, and studies not meeting the inclusion criteria or meeting exclusion criteria were removed. Extracted information included study characteristics (title, authors, year, design, diagnostic criteria, interventions, treatment duration, outcomes), participant demographics (sample size, age, sex), and methodological details (randomization, allocation concealment, blinding). Disagreements at any stage were resolved through discussion, with a third researcher consulted when necessary. When essential details were missing, study authors were contacted for clarification.

### Risk of bias and quality assessment

Two researchers independently assessed the risk of bias in the included studies following the Cochrane Handbook (20). The evaluation covered sequence generation, allocation concealment, blinding, completeness of outcome data, selective reporting, and other potential biases. Disagreements were resolved through discussion or consultation with a third researcher. In addition, the overall confidence in the network meta-analysis was appraised using the CINeMA (Confidence in Network Meta-Analysis) framework, which evaluates within-study bias, reporting bias, indirectness, imprecision, heterogeneity, and incoherence.

### Statistical analysis

We performed network meta-analyses of RCTs using Stata version 17.0 within a frequentist framework. Relative risks (RRs) with 95% confidence intervals (CIs) were used to compare dichotomous outcomes, including the total effective rate. Odds ratios (ORs) with 95% CIs were applied for adverse event rates, given their suitability in analyzing rare events. Mean differences (MDs) with 95% CIs were used for continuous outcomes, such as MTL and GAS levels. Random-effects models were applied using the restricted maximum likelihood (REML) method to account for between-study heterogeneity.

Heterogeneity was assessed with τ^2^, with thresholds defined as low (<0.04), low—moderate (0.04–0.16), moderate—high (0.16–0.36), and high (>0.36), according to published recommendations. The τ^2^ value was assumed constant across all comparisons, and a correlation of 0.5 was assumed in the between-study covariance matrix. We used the node-splitting method to examine local consistency between direct and indirect comparisons in closed loops of evidence, and a design-by-treatment interaction model to evaluate global inconsistency across the network. Network diagrams were generated in Stata, in which each node represented an intervention and connecting lines denoted direct comparisons, with the size of nodes and thickness of edges weighted by the number of patients and studies. To compare the relative efficacy of interventions, we constructed a league table of pairwise comparisons and calculated the surface under the cumulative ranking curve (SUCRA) to rank treatments. SUCRA values range from 0 (least effective) to 1 (most effective) and were interpreted with caution, taking into account the clinical relevance of observed differences.

## Results

### Literature screening results

**Figure 1:**
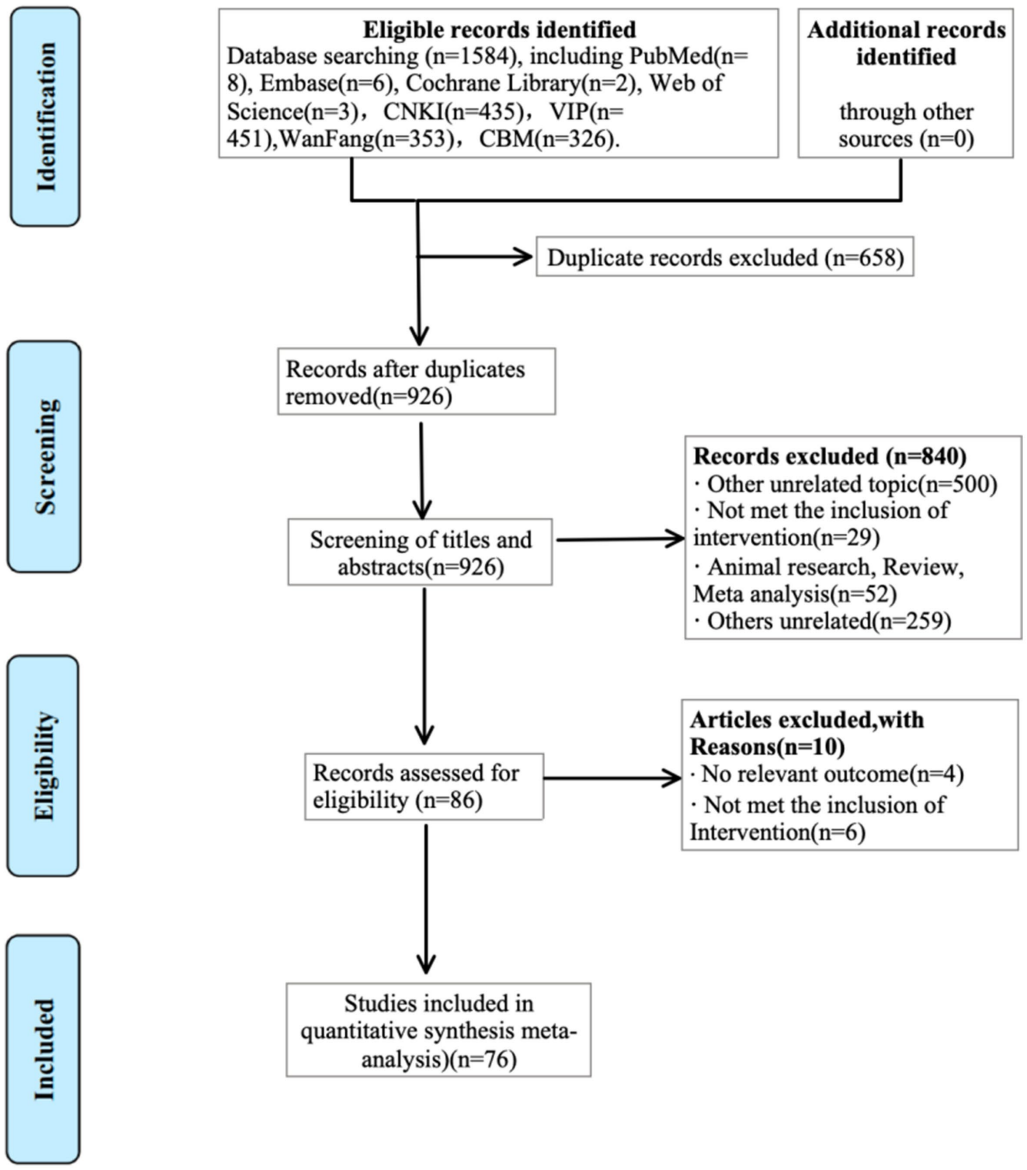
Flowchart of literature search.

A total of 1,584 records were retrieved from PubMed (*n* = 8), Embase (*n* = 6), the Cochrane Library (*n* = 2), Web of Science (*n* = 3), CNKI (*n* = 435), VIP (*n* = 451), WanFang (*n* = 353), and CBM (*n* = 326). After removing 658 duplicates, 926 records remained for screening. Based on titles and abstracts, 840 records were excluded as irrelevant, non-randomised, or animal studies. Of the 86 articles reviewed in full, 10 were excluded for reasons including lack of relevant outcomes (*n* = 4) or ineligible interventions (*n* = 6). Ultimately, 76 RCTs met the eligibility criteria and were included in the quantitative synthesis. The study selection process is illustrated in Figure 1.

### Basic features of the included literature

Seventy-six studies were ultimately included in this analysis, comprising a combined sample size of 7,575 participants, with 3,805 in the intervention groups and 3,770 in the control groups. The number of RCTs involving various interventions included are as follows: 14 RCTs on ZKC + CWM (21–34), 6 on WLC + CWM (35–40), 5 on QWG + CWM (41–45), 5 on XPG + CWM (46–50), 4 on DLTG + CWM (51–54), 11 on WSG + CWM (55–65), 3 on JWC + CWM (66–68), 5 on WCAC + CWM (69–73), 3 on BWC + CWM (74–76), 8 on STOL + CWM (77–84), 6 on LNC + CWM (85–90), and 6 on LAC + CWM (91–96). The basic characteristics of these studies are summarized in Table 1.

**Table 1:** Basic characteristics of the included studies.

Study ID	Sample size (T/C)	Age (year, T/C)	Disease duration (T/C)	Duration of treatment	Treatment group	Control group	CWM regimen	Outcomes
Liu 2024 (21)	51/51	44.59 ± 9.60/44.52 ± 9.64	3.55 ± 0.27/3.51 ± 0.24a	1 m	ZKC + CWM	CWM	Mosapride citrate 5 mg TID	①②③④
Yang 2024 (22)	46/46	43.42 ± 9.46/43.46 ± 9.51	5.06 ± 1.78/5.09 ± 1.74a	1 m	ZKC + CWM	CWM	Flupentixol-melitracen 1 tab QD	①②③④
Yin 2021 (23)	42/42	47.98 ± 9.79/48.37 ± 10.15	5.63 ± 1.82/5.46 ± 1.71a	1 m	ZKC + CWM	CWM	Erythromycin enteric-coated 0.5 g BID	①④
Wang 2020 (24)	68/67	41.02 ± 6.01/40.51 ± 6.09	1.90 ± 0.39/1.96 ± 0.41a	2 w	ZKC + CWM	CWM	Esomeprazole + Itopride 50 mg TID	①②③
Wang 2020 (25)	30/30	58.64 ± 11.57/57.32 ± 11.03	5.43 ± 0.86/4.98 ± 0.87a	1 m	ZKC + CWM	CWM	Mosapride 5 mg TID	①②
Huang 2019 (26)	37/37	47.00 ± 6.32/48.00 ± 6.35	1.50 ± 0.28/1.35 ± 0.26a	1 m	ZKC + CWM	CWM	Mosapride 5 mg TID	①④
Li 2019 (27)	48/48	64.76 ± 2.15/62.16 ± 3.42	1.6 ± 1.1/1.8 ± 0.9a	1 m	ZKC + CWM	CWM	Mosapride 5 mg TID	②③
Wu 2018 (28)	37/37	43.67 ± 4.61/44.57 ± 5.13	4.59 ± 1.24/4.38 ± 1.30a	1 m	ZKC + CWM	CWM	Mosapride citrate 5 mg TID	①②③④
Yang 2018 (29)	42/41	35.68 ± 11.31/36.23 ± 12.71	−/−	1 m	ZKC + CWM	CWM	Domperidone 10 mg TID	①②③④
Li 2018 (30)	62/62	46.28 ± 7.16/46.23 ± 7.25	1.74 ± 0.26/1.77 ± 0.24a	1 m	ZKC + CWM	CWM	Mosapride 5 mg TID	①②
He 2017 (31)	41/41	46.1 ± 4.5/46.2 ± 4.6	−/−	1 m	ZKC + CWM	CWM	Mosapride 5 mg TID	①②④
Wang 2015 (32)	66/66	46.25 ± 4.67/46.88 ± 5.31	2.54 ± 0.15/2.21 ± 0.14a	1 m	ZKC + CWM	CWM	Mosapride 5 mg TID	①②④
Yuan 2012 (33)	40/40	39.5 ± 4.3/37.5 ± 4.8	15.5 ± 4.5/14.7 ± 3.8a	1 m	ZKC + CWM	CWM	Domperidone 10 mg TID	①
Lei 2012 (34)	42/40	22 ~ 55	−/−	2 w	ZKC + CWM	CWM	Omeprazole 10 mg BID	①④
Zhang 2021 (35)	38/38	61.84 ± 2.31/62.17 ± 2.14	4.14 ± 1.35/4.04 ± 1.64a	2 w	WLC + CWM	CWM	Domperidone 10 mg TID	②
Gu 2016 (36)	100/100	45.3 ± 10.1/44.7 ± 10.7	2.1 ± 0.4/2.3 ± 0.6a	2 m	WLC + CWM	CWM	Itopride 50 mg TID	①②③
Wang 2015 (37)	52/52	48.7 ± 10.4/49.5 ± 11.0	2.9 ± 0.7/3.1 ± 0.9a	1 m	WLC + CWM	CWM	Bifidobacterium triple viable (Bifico) 2 caps TID	①④
Feng 2014 (38)	30/30	40/41	−/−	1 m	WLC + CWM	CWM	Mosapride citrate 5 mg TID	①
Zhang 2012 (39)	45/45	43.8 ± 15.0/43.5 ± 11.6	−/−	1 m	WLC + CWM	CWM	Itopride 50 mg TID	①④
Li 2011 (40)	80/70	43.3 ± 10.2/40 ± 10.3	−/−	2 m	WLC + CWM	CWM	Trimebutine maleate 100 mg TID	①④
Wei 2018 (41)	40/40	39.65 ± 1.32/39.32 ± 1.28	10.8 ± 0.7/10.5 ± 0.5 m	1 m	QWG + CWM	CWM	Mosapride 5 mg TID	①④
Xu 2017 (42)	60/60	39.8 ± 1.4/40.2 ± 1.3	11.5/11.3 m	1 m	QWG + CWM	CWM	Mosapride 5 mg TID	①②
Zhang 2017 (43)	40/40	40.6 ± 19.9/41.5 ± 18.6	5.5 ± 4.2/5.0 ± 4.8a	2 w	QWG + CWM	CWM	Omeprazole 10 mg QD	①④
Gu 2015 (44)	35/35	39.5/38.4	11.3/11.5 m	1 m	QWG + CWM	CWM	Mosapride 5 mg TID	①②
Chen 2014 (45)	46/46	43.8 ± 5.1/42.3 ± 5.4	12.8 ± 3.8/13.4 ± 3.6a	1 m	QWG + CWM	CWM	Domperidone 10 mg TID	①
Lin 2023 (46)	43/43	41.50 ± 7.80/42.10 ± 7.50	15.2 ± 2.2/15.3 ± 4.2 m	1 m	XPG + CWM	CWM	Mosapride citrate 5 mg TID	①②③④
Li 2022 (47)	35/35	42.45 ± 4.57/42.15 ± 6.85	1.91 ± 0.83/1.86 ± 0.79a	1 m	XPG + CWM	CWM	Digestive enzyme compound 1 tab TID	②③④
Zhang 2021 (48)	42/41	40.4 ± 5.1/40.7 ± 4.8	10.7 ± 2.7/10.8 ± 2.6a	1 m	XPG + CWM	CWM	Itopride 50 mg TID	①②③
Zhou 2019 (49)	53/53	46.73 ± 4.28/46.18 ± 4.75	12.86 ± 4.0/13.07 ± 4.4a	1 m	XPG + CWM	CWM	Mosapride citrate 5 mg TID	①②③④
Wang 2018 (50)	50/50	36.58 ± 10.4/35.99 ± 10.	13.65 ± 3.2/13.98 ± 3.5a	1 m	XPG + CWM	CWM	Trimebutine maleate 200 mg TID	①②③
Luo 2023 (51)	41/41	43.27 ± 2.13/43.87 ± 2.53	25.41 ± 5.1/25.94 ± 5.5a	2 w	DLTG+CWM	CWM	Dallitong granules 6 g TID	①②③
Hu 2021 (52)	44/44	−/−	−/−	1 m	DLTG+CWM	CWM	Itopride 50 mg TID	①②③④
Cai 2019 (53)	42/42	43.90 + 5.96/43.45 + 5.36	3.45 ± 1.09/3.30 ± 0.90a	1 m	DLTG+CWM	CWM	Itopride 50 mg TID	①②③
Zhu 2011 (54)	45/45	41/40	−/−	1 m	DLTG+CWM	CWM	Trimebutine maleate 100 mg TID	①
Gao 2022 (55)	50/50	37.74 ± 6.42/37.82 ± 6.35	5.68 ± 1.18/5.62 ± 1.24a	1 m	WSG + CWM	CWM	*Bacillus licheniformis* capsule 0.25 g, 2 caps TID (first dose double)	①④
Li 2022 (56)	60/60	54.24 ± 9.82/53.68 ± 7.96	3.16 ± 0.87/3.09 ± 0.90a	1 m	WSG + CWM	CWM	Itopride 50 mg TID	①②③
Zhou 2021 (57)	47/46	36.14 ± 3.51/36.28 ± 3.54	6.26 ± 3.09/6.23 ± 3.10a	1 m	WSG + CWM	CWM	Mosapride 5 mg TID	①②③④
Liang 2021 (58)	46/47	42.26 ± 2.33/41.79 ± 2.58	2.93 ± 0.47/2.96 ± 0.52a	1 m	WSG + CWM	CWM	Domperidone 10 mg TID	①②③④
Lin 2021 (59)	40/40	44.35 ± 6.36/46.52 ± 7.19	5.12 ± 4.08/4.89 ± 3.21a	1 m	WSG + CWM	CWM	Mosapride 5 mg TID	①④
Zhang 2018 (60)	130/130	−/−	−/−	1 m	WSG + CWM	CWM	Mosapride 5 mg TID	①
Zabila 2018 (61)	45/45	44.23 ± 6.89/43.78 ± 7.23	−/−	1 m	WSG + CWM	CWM	Mosapride 5 mg TID	①
Zhao 2017 (62)	80/70	43.26 ± 10/44.19 ± 10	−/−	1 m	WSG + CWM	CWM	Mosapride 5 mg TID	①
Liu 2017 (63)	25/20	44.2 ± 6.8/43.7 ± 7.2	−/−	1 m	WSG + CWM	CWM	Mosapride 5 mg TID + Compound digestive enzyme 1–2 caps TID	①
Zhang 2016 (64)	40/40	45.32 ± 9/42.67 ± 10	3.23 ± 1.94/4.20 ± 2.32a	1 m	WSG + CWM	CWM	Mosapride 5 mg TID	①
Zhang 2012 (65)	33/33	29.4/29.2	−/−	1 m	WSG + CWM	CWM	Domperidone 10 mg TID	①
Sun 2014 (66)	31/30	36 ± 5/37 ± 5	−/−	1 m	JWC + CWM	CWM	Trimebutine 100 mg TID before or	①
Lu 2014 (67)	60/60	42.7 ± 2.5/44.7 ± 1.6	−/−	1 m	JWC + CWM	CWM	Itopride hydrochloride 50 mg TID 30 min	①
Wang 2014 (68)	79/79	51.0 ± 12.1/48.1 ± 13.5	3.26 ± 2.14/3.12 ± 2.06a	1 m	JWC + CWM	CWM	Itopride hydrochloride 50 mg TID 30 min	①④
Hu 2023 (69)	41/41	6.72 ± 2.55/6.69 ± 2.60	3.21 ± 0.8/3.17 ± 0.9 m	1 m	WCAC+CWM	CWM	Omeprazole 0.8–1.0 mg/kg BID + Amoxicillin 20–40 mg/kg TID + Clarithromycin 7.5 mg/kg BID	①④
Kong 2020 (70)	46/46	5.5 ± 2.0/5.0 ± 1.7	5.5 ± 2.0/5.0 ± 1.7 m	2 w	WCAC+CWM	CWM	Mosapride 5 mg TID	①
Wei 2019 (71)	50/50	45.20 ± 12.4/47.12 ± 10.9	5.32 ± 1.32/5.51 ± 1.74a	2 w	WCAC+CWM	CWM	Domperidone 10 mg TID	①②③
Zhou 2018 (72)	55/55	6.4 ± 1.7/6.6 ± 1.5	6.3 ± 1.2/6.1 ± 1.4a	1 m	WCAC+CWM	CWM	Domperidone 0.3 mg/kg TID	①②
Zhi 2017 (73)	51/51	6.52 ± 1.10/6.61 ± 1.25	8.14 ± 1.23/8.65 ± 1.20a	1 m	WCAC+CWM	CWM	Domperidone 0.3 mg/kg TID + Omeprazole 0.8–1.0 mg/kg BID + Amoxicillin 20–40 mg/kg TID + Clarithromycin 15–20 mg/kg BID	①②④
Zhang 2023 (74)	45/45	43.83 ± 9.97/44.81 ± 9.31	8.37 ± 2.9/8.99 ± 2.9 m	1 m	BWC + CWM	CWM	Pancreatin (220 mg) TID with meals	①②③④
Zhang 2022 (75)	60/60	48.63 ± 2.95/48.90 ± 2.74	3.40 ± 1.18/3.49 ± 1.27a	1 m	BWC + CWM	CWM	Mosapride 5 mg TID + Rabeprazole 10 mg BID	①
Jin 2020 (76)	53/53	67.51 ± 5.40/67.09 ± 6.22	17.61 ± 3/17.34 ± 3 m	1 w	BWC + CWM	CWM	Mosapride citrate 5 mg TID	①④
Zhao 2021 (77)	57/57	7.01 ± 1.65/6.59 ± 1.58	12.13 ± 4/12.52 ± 4 m	2 w	STOL+CWM	CWM	Saccharomyces boulardii powder 250 mg QD (<3 y) or BID (>3 y),	①②③④
Gao 2018 (78)	100/100	5.86 ± 0.53/5.78 ± 0.45	1.72 ± 1.3/2.80 ± 0.3a	2 w	STOL+CWM	CWM	NSAIDs + prokinetics (routine Western therapy, unspecified)	①②
Fu 2018 (79)	50/50	46.9 ± 19.1/53.7 ± 15.9	−/−	2 w	STOL+CWM	CWM	Mosapride 5 mg TID	①
Wang 2018 (80)	30/30	7.7 ± 1.8/7.8 ± 1.5	−/−	2 w	STOL+CWM	CWM	Domperidone 0.3 mg/kg TID	①
Zou 2016 (81)	42/42	45.75 ± 8.52/43.49 ± 9.18	5.45 ± 2.7/4.86 ± 2.9 m	2 w	STOL+CWM	CWM	Compound azintamide 2 tablets TID	①
Qi 2016 (82)	57/57	2.35 ± 1.08/2.46 ± 1.13	−/−	1 w	STOL+CWM	CWM	Bifidobacterium triple viable capsule 630 mg TID	①②③
Zhang 2016 (83)	46/46	45.1 ± 5.2/44.6 ± 4.9	16.7 ± 3.2/17.3 ± 3.5 m	2 m	STOL+CWM	CWM	Bifidobacterium triple viable capsule 630 mg TID	①
Meng 2014 (84)	50/50	2.5 ± 1.5/2.6 ± 1.7	−/−	1 w	STOL+CWM	CWM	Bifidobacterium triple viable capsule 630 mg TID	①②③
Xu 2021 (85)	35/35	31.25 ± 3.64/31.34 ± 3.71	12.5 ± 3.6/12.6 ± 3.7 m	2 w	LNC + CWM	CWM	Lactobacillus tablets 0.6 g TID, Mosapride citrate 5 mg TID	②③④
Xing 2021 (86)	40/40	39.71 ± 5.66/39.96 ± 5.25	1.55 ± 0.71/1.51 ± 0.73a	1 m	LNC + CWM	CWM	Compound digestive enzyme capsule 2 capsules TID	①②③④
Wu 2020 (87)	83/83	47.5 ± 2.3/48.5 ± 2.2	3.2 ± 0.6/3.6 ± 0.5a	1 m	LNC + CWM	CWM	Compound azintamide enteric-coated tablet 1–2 tablets TID	①②③④
Chen 2019 (88)	35/35	42.47 ± 13.2/41.83 ± 13.4	−/−	1 m	LNC + CWM	CWM	Flupentixol-melitracen tablet 1 tablet QD	①②③④
Zhang 2017 (89)	56/56	58.5 ± 2.5/56.8 ± 2.8	−/−	2 m	LNC + CWM	CWM	Mosapride 5 mg TID	①
Yang 2012 (90)	35/31	−/−	−/−	1 m	LNC + CWM	CWM	Compound digestive enzyme capsule 2 capsules TID	①
Liu 2020 (91)	50/50	47.41 ± 6.14/48.54 ± 6.45	3.74 ± 1.53/3.53 ± 1.32a	1 m	LAC+CWM	CWM	Lansoprazole 30 mg QD	①④
Du 2019 (92)	74/74	48.36 ± 10.4/47.95 ± 10.1	3.17 ± 2.09/3.29 ± 2.31a	2 m	LAC+CWM	CWM	Omeprazole 20 mg BID	①②
Gao 2019 (93)	50/50	22 ~ 59/23 ~ 60	−/−	1 m	LAC+CWM	CWM	Itopride 50 mg TID	①
Wang 2013 (94)	60/60	39.6 ± 9.2/40.3 ± 9.8	−/−	1 m	LAC+CWM	CWM	Domperidone 10 mg TID	①
Yang 2012 (95)	45/45	39.6 ± 9.2/40.3 ± 9.8	0.68/0.48.5a	1 m	LAC+CWM	CWM	Compound digestive enzyme capsule 2 capsules TID	①
Wang 2012 (96)	30/30	−/−	−/−	1 m	LAC+CWM	CWM	Domperidone 10 mg TID, Potassium bismuth citrate granules 110 mg TID	①

T, treatment group; C, control group; −, not report; ① Total effective rate; ② levels of motilin (MTL); ③ levels of gastrin (GAS); ④ Adverse event rate; ZKC, Zhizhu Kuanzhong Capsules; WLC, Wuling Capsules; QWG, Qizhi Weitong Granules; XPG, Xiangsha Pingwei Granules; DLTG, Dalitong Granules; WSG, Weisu Granules; JWC, Jinghua Weikang Capsules; WCAC, Weichang An Capsules; BWC, Biling Weitong Granules; STOL, Simo Tang Oral Liquid; LNC, Liuwei Nengxiao Capsules; LAC, Liuwei Anxiao Capsules; CWM, conventional Western medicine. a, year; m, month; w, week.

### Bias analysis and evaluation

**Figure 2:**
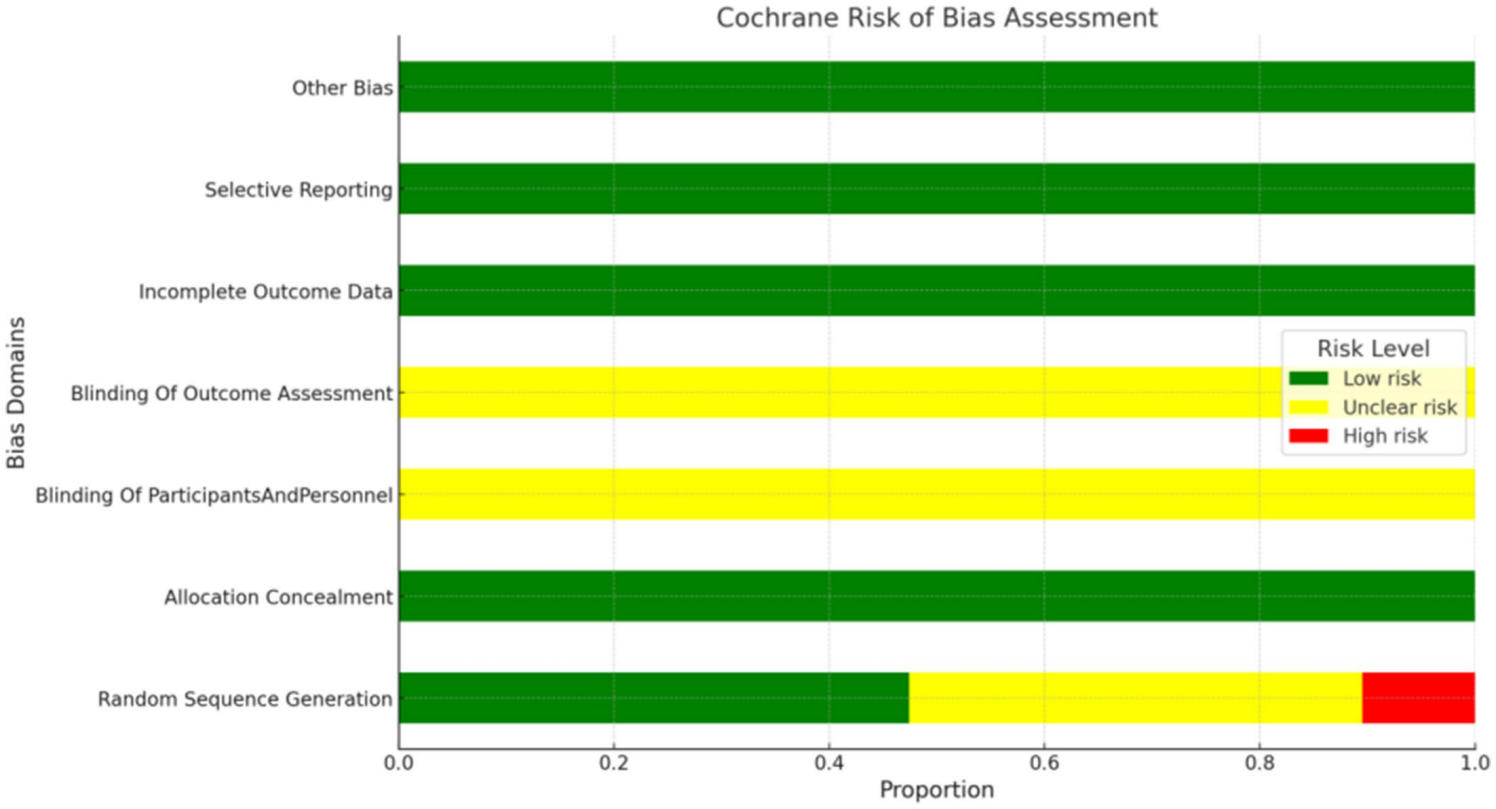
Risk of bias graph.

Thirty-six studies were rated as “Low risk” for explicitly mentioning their random number generation methods (e.g., random number tables, drawing lots). In contrast, 32 studies were rated as “Unclear risk” due to merely mentioning random allocation without specifying the methods used. Eight studies were rated as “High risk” for either not mentioning random allocation or using non-random methods for grouping (such as assignment by date of admission, order of clinic visit, or date of birth). All studies were rated as “Unclear risk” due to a lack of mention of concealment in the allocation process. However, the studies did not mention blinding and were thus rated as “Unclear risk.” All studies were rated as “Low risk” for data completeness and the absence of selective reporting. Due to limited information, assessing other potential biases in the included literature was impossible, so they were rated as “Unclear risk.” The risk of bias assessment is depicted in Figure 2.

### Certainty of evidence and consistency

To evaluate the consistency between direct and indirect evidence, we performed a network meta-analysis under a frequentist framework. The consistency model showed good agreement across all comparisons, with no significant inconsistency detected (*p* > 0.05). Heterogeneity, assessed using the τ^2^ statistic, was generally low to moderate, supporting the reliability of the pooled estimates (Appendix 4). Using CINeMA to evaluate confidence in the evidence, most pairwise comparisons were rated as low (Appendix 7). All networks satisfied the transitivity assumption, ensuring the validity of indirect comparisons (Appendix 7, Table S5.1). In addition, funnel plots revealed no evidence of asymmetry (Appendix 8).

### Total effective rate

**Figure 3:**
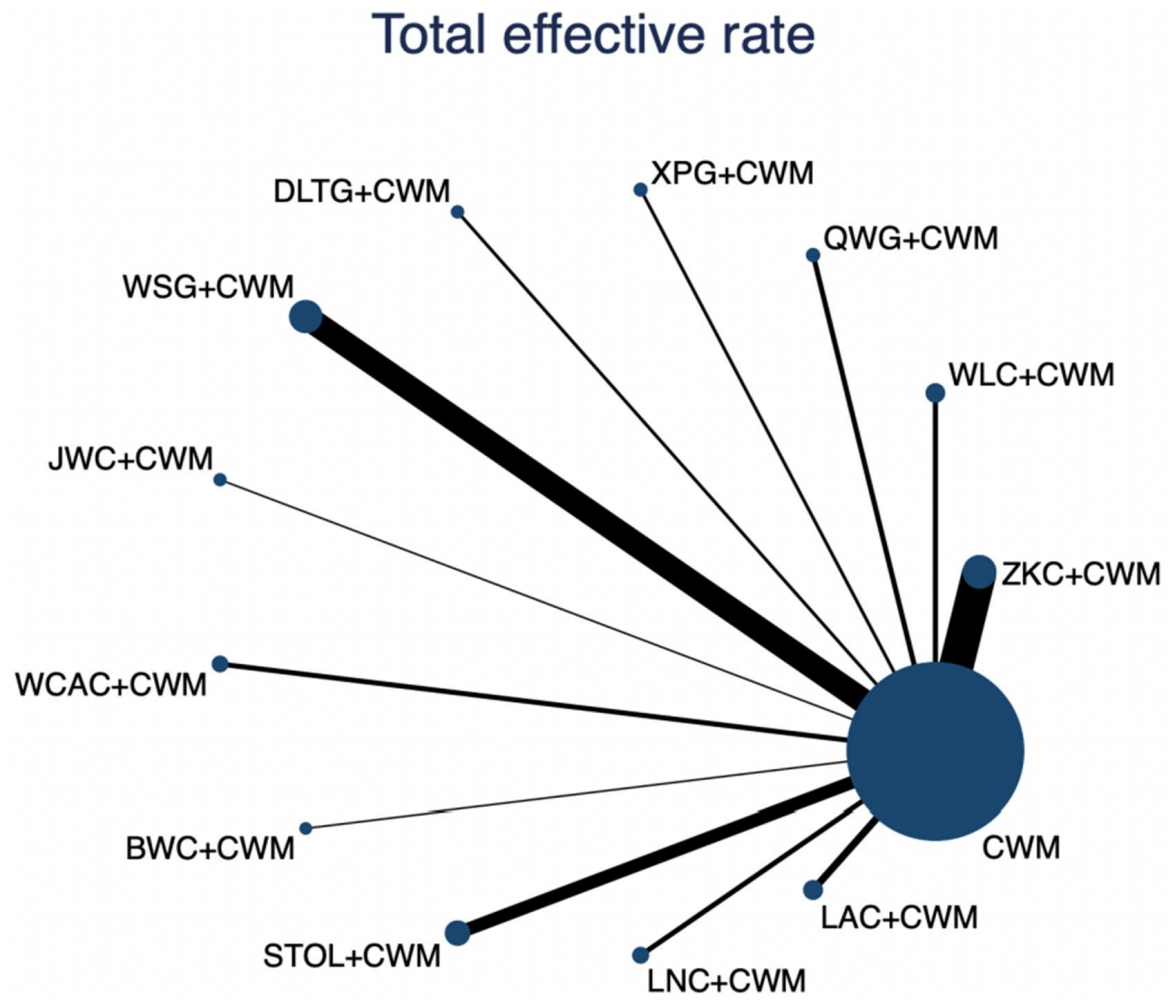
Network of available comparisons for total effective rate. The size of each node is proportional to the total number of randomized participants allocated to that intervention, and the thickness of the connecting lines reflects the number of participants included in head-to-head trials. Interventions include CWM alone and CWM combined with one of twelve CPMs: Zhizhu Kuanzhong Capsules (ZKC), Wuling Capsules (WLC), Qizhi Weitong Granules (QWG), Xiangsha Pingwei Granules (XPG), Dalitong Granules (DLTG), Weisu Granules (WSG), Jinghua Weikang Capsules (JWC), Weichang An Capsules (WCAC), Bilin Weitong Granules (BWC), Simo Tang Oral Liquid (STOL), Liuwei Nengxiao Capsules (LNC), and Liuwei Anxiao Capsules (LAC).

**Figure 4:**
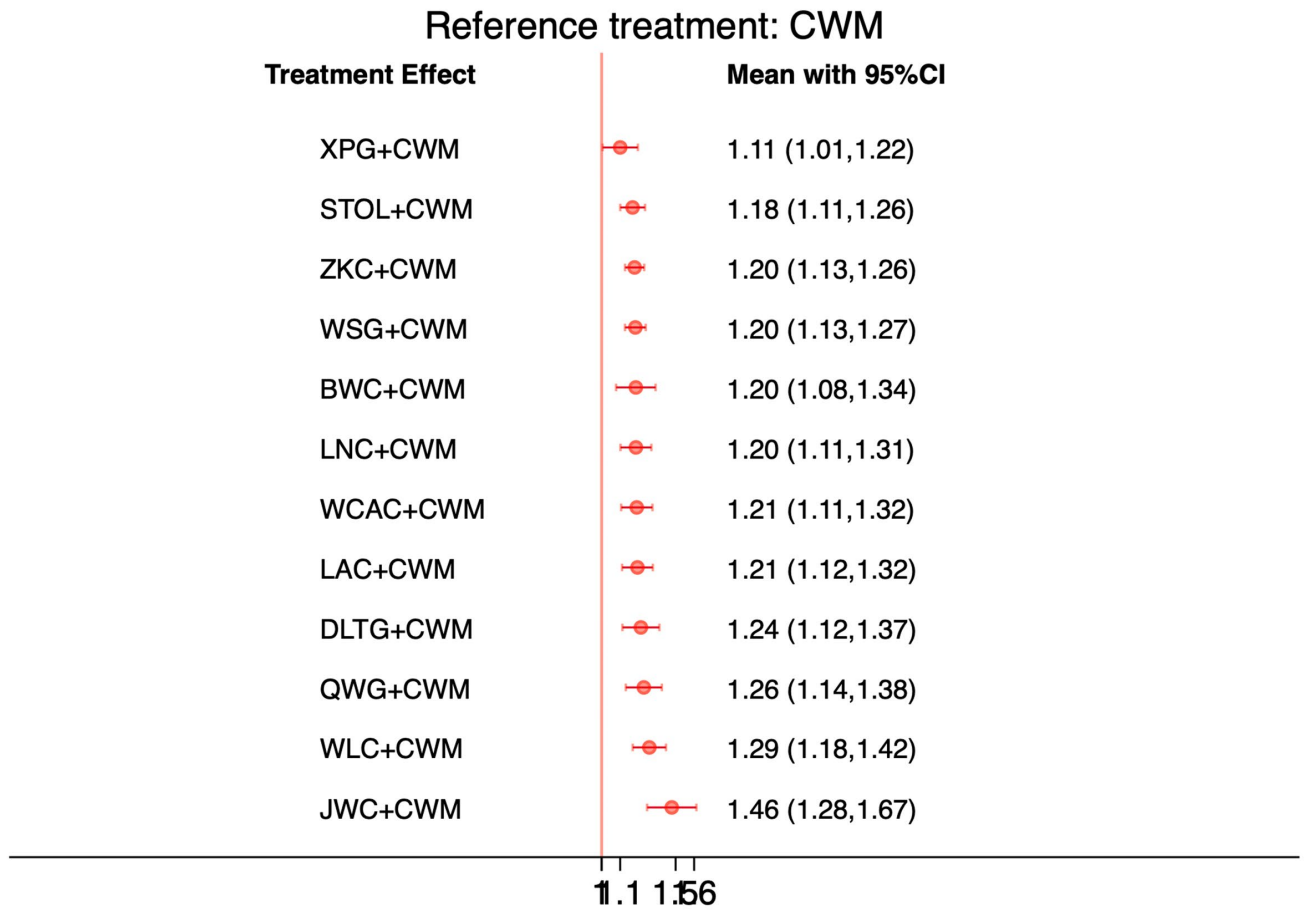
Forest plot of network meta-analysis comparing CPMs combined with CWM versus CWM alone for total effective rate. Effect sizes are expressed as RRs with 95% confidence intervals, using CWM as the common reference treatment. Values to the right of the vertical line indicate a higher probability of clinical effectiveness compared with CWM.

**Figure 5:**
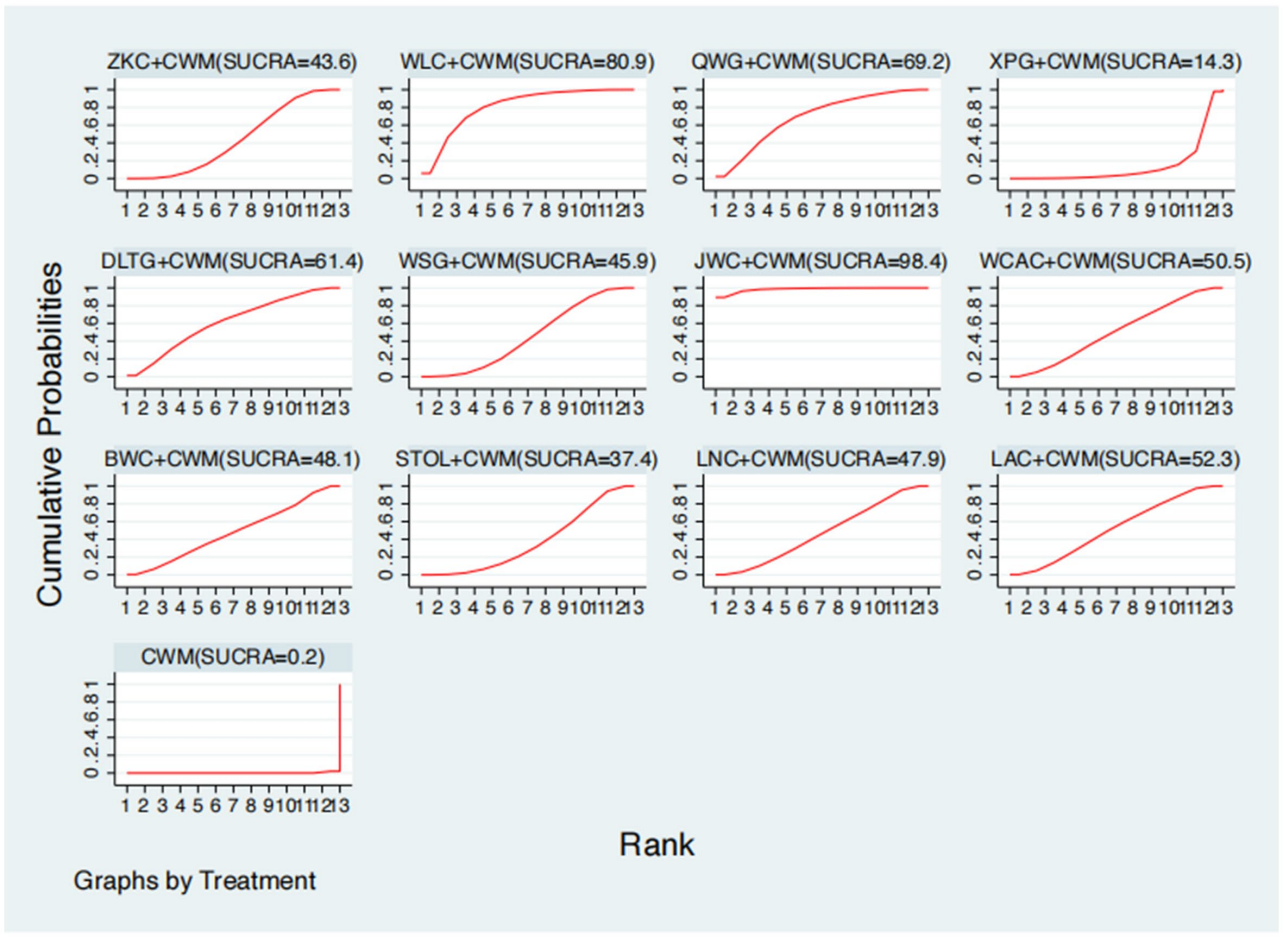
Surface under the cumulative ranking curve (SUCRA) for total effective rate.

This network meta-analysis included 72 RCTs involving 7,186 participants who reported data on total effective rate. The network plot demonstrated that all 12 CPMs combined with CWM were compared, directly or indirectly, with CWM alone (Figure 3). Node size was proportional to the number of participants in each intervention, and line thickness reflected the number of trials directly comparing two treatments. Overall, the forest plot indicated that all CPM + CWM regimens were more effective than CWM alone, with risk ratios consistently greater than 1 and 95% confidence intervals not crossing the null (Figure 4). The most substantial benefit was observed for JWC + CWM (RR = 1.46; 95% CI 1.28–1.67; SUCRA = 98.4%), followed by WLC + CWM (RR = 1.29; 95% CI 1.18–1.42; SUCRA = 80.9%) and QWG + CWM (RR = 1.26; 95% CI 1.14–1.38; SUCRA = 69.2%) (Figure 5). Head-to-head comparisons among CPM regimens further supported the superiority of JWC + CWM. JWC + CWM was significantly more effective than WCAC + CWM (RR = 1.21; 95% CI 1.03–1.41), BWC + CWM (RR = 1.21; 95% CI 1.02–1.44), STOL + CWM (RR = 1.24; 95% CI 1.06–1.43), LNC + CWM (RR = 1.21; 95% CI 1.04–1.42), and LAC + CWM (RR = 1.20; 95% CI 1.03–1.41). In contrast, XPG + CWM was less effective than JWC + CWM (RR = 0.76; 95% CI 0.64–0.89). When comparing WLC + CWM and XPG + CWM, the former showed a statistically significant advantage (RR = 1.17; 95% CI 1.03–1.33). The complete league table of relative effect estimates is presented in Appendix 6 (Table S1).

### Serum MTL levels

**Figure 6:**
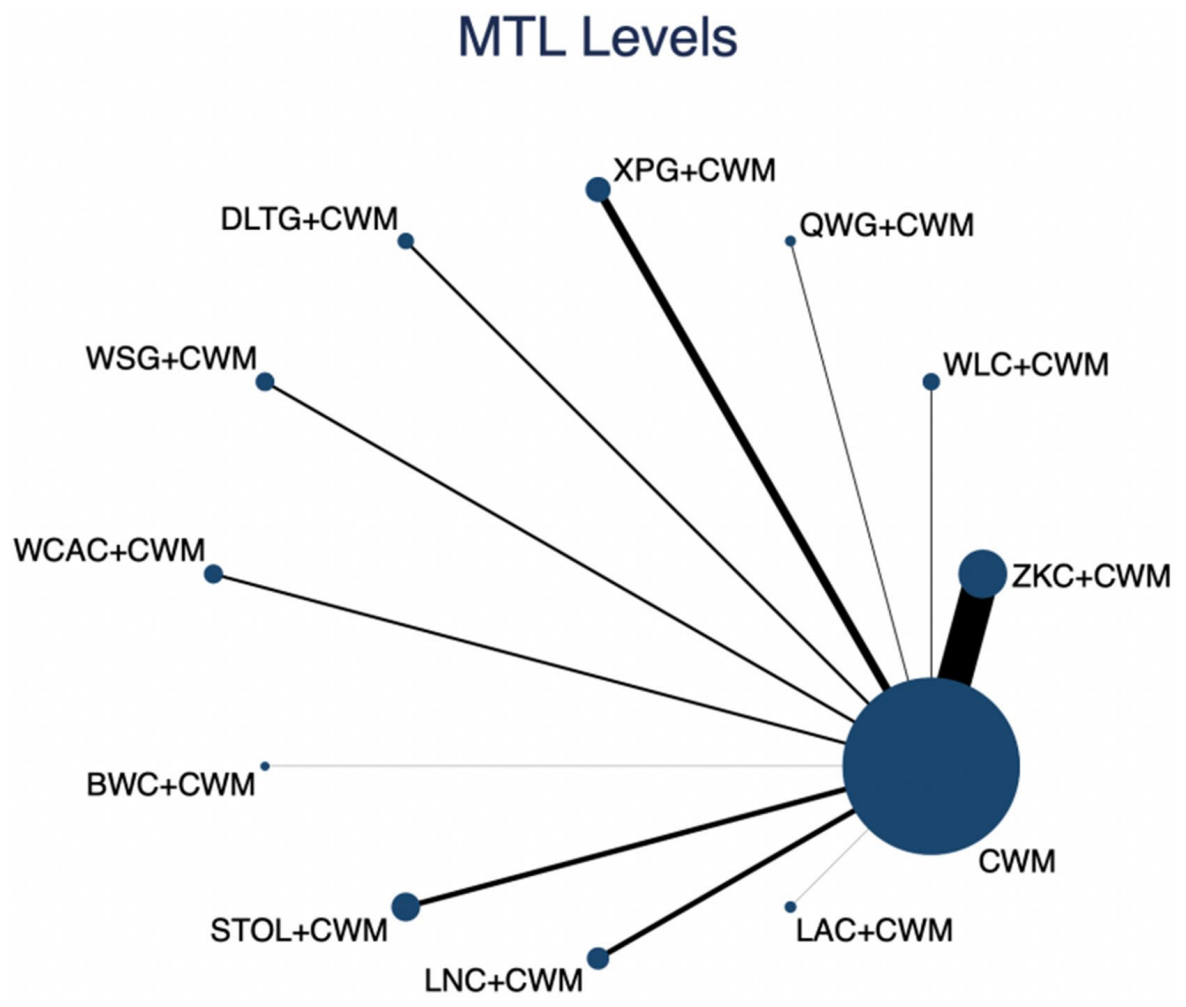
Network of available comparisons for motilin (MTL) levels. The size of each node is proportional to the total number of randomized participants allocated to that intervention, and the thickness of the connecting lines reflects the number of participants included in head-to-head trials. Interventions include CWM alone and CWM combined with one of twelve CPMs: Zhizhu Kuanzhong Capsules (ZKC), Wuling Capsules (WLC), Qizhi Weitong Granules (QWG), Xiangsha Pingwei Granules (XPG), Dalitong Granules (DLTG), Weisu Granules (WSG), Jinghua Weikang Capsules (JWC), Weichang An Capsules (WCAC), Bilin Weitong Granules (BWC), Simo Tang Oral Liquid (STOL), Liuwei Nengxiao Capsules (LNC), and Liuwei Anxiao Capsules (LAC).

**Figure 7:**
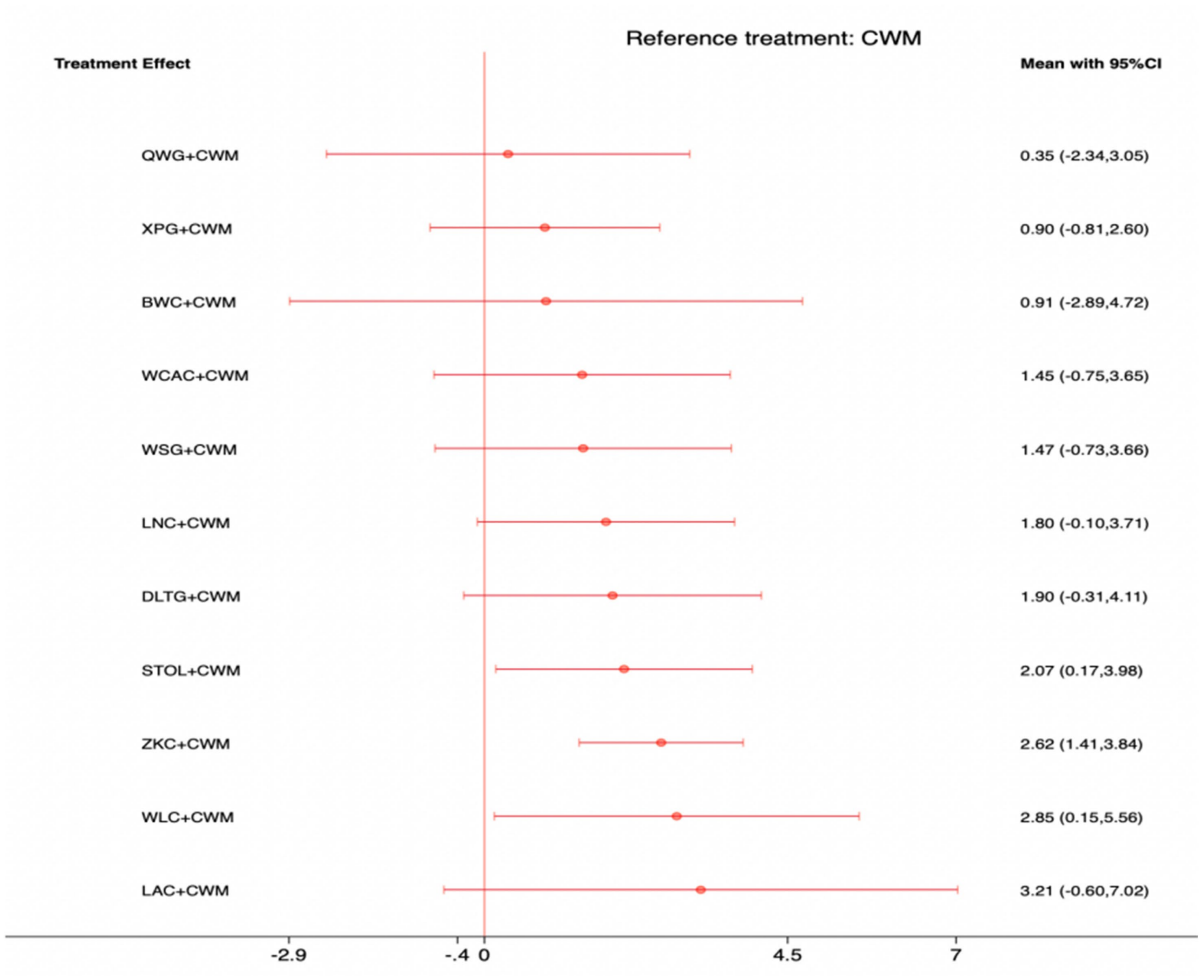
Forest plot of network meta-analysis comparing CPMs combined with CWM versus CWM alone for motilin (MTL) levels. Effect sizes are expressed as mean differences (MDs) with 95% confidence intervals, using CWM as the common reference treatment. Values to the right of the vertical line indicate higher MTL levels compared with CWM.

**Figure 8:**
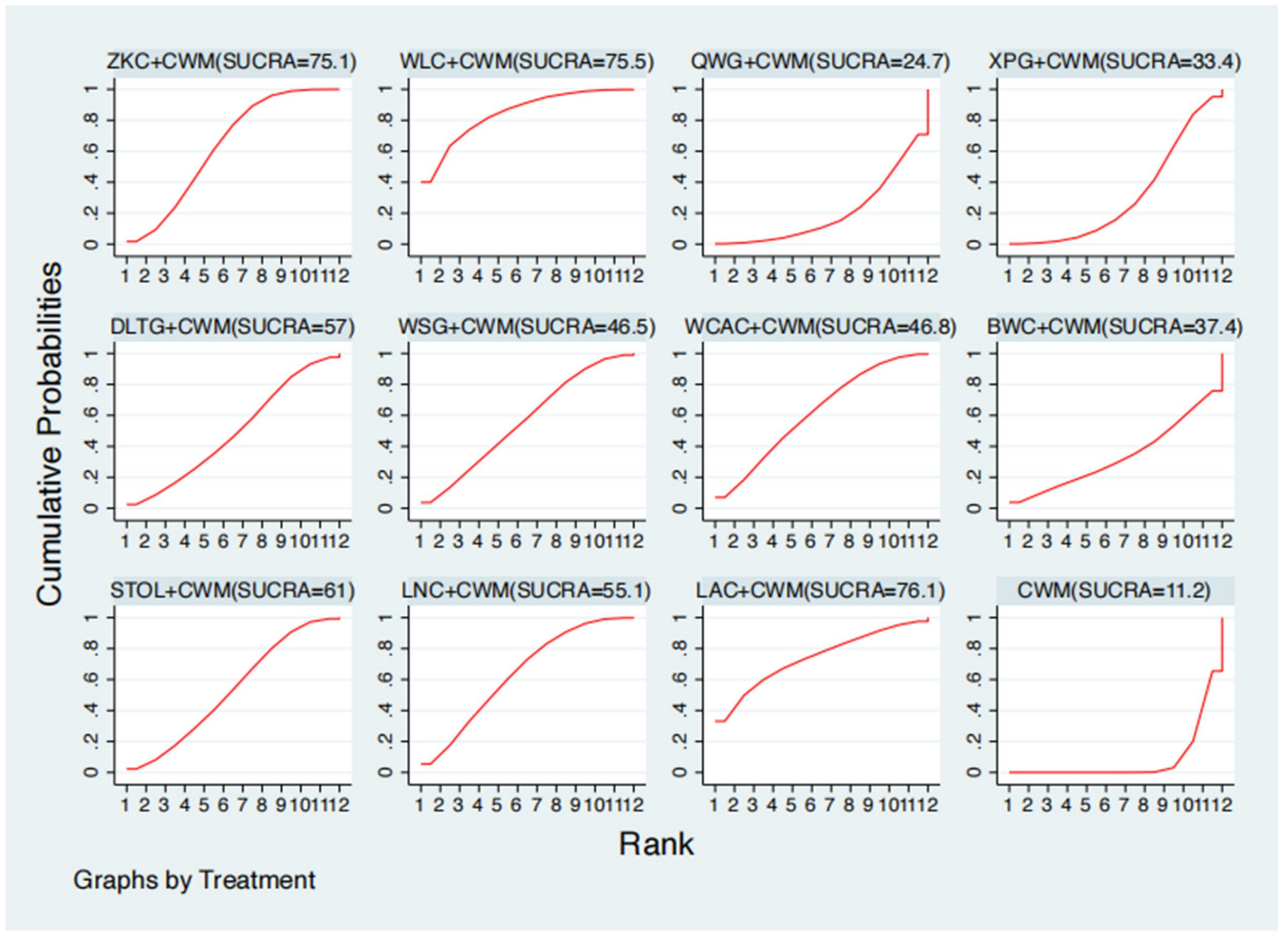
Surface under the cumulative ranking curve (SUCRA) for motilin (MTL) levels.

This network meta-analysis included 38 RCTs involving 3,848 participants who reported data on MTL levels. The network plot demonstrated that all 11 CPMs combined with CWM were compared, directly or indirectly, with CWM alone (Figure 6). Node size was proportional to the number of participants in each intervention, and line thickness reflected the number of trials directly comparing two treatments. Overall, the forest plot indicated that several CPM + CWM regimens were associated with significantly increased MTL levels compared with CWM alone (Figure 7). The most substantial improvements were observed with WLC + CWM (MD = 2.85; 95% CI 0.15–5.56; SUCRA = 75.5%), followed by ZKC + CWM (MD = 2.62; 95% CI 1.41–3.84; SUCRA = 75.1%), and STOL + CWM (MD = 2.07; 95% CI 0.17–3.98; SUCRA = 61.0%) (Figure 8). Indirect comparisons among CPM regimens based on the league table revealed no statistically significant differences between individual CPM + CWM combinations. The complete league table of relative effect estimates is presented in Appendix 6 (Table S2).

### Serum GAS levels

**Figure 9:**
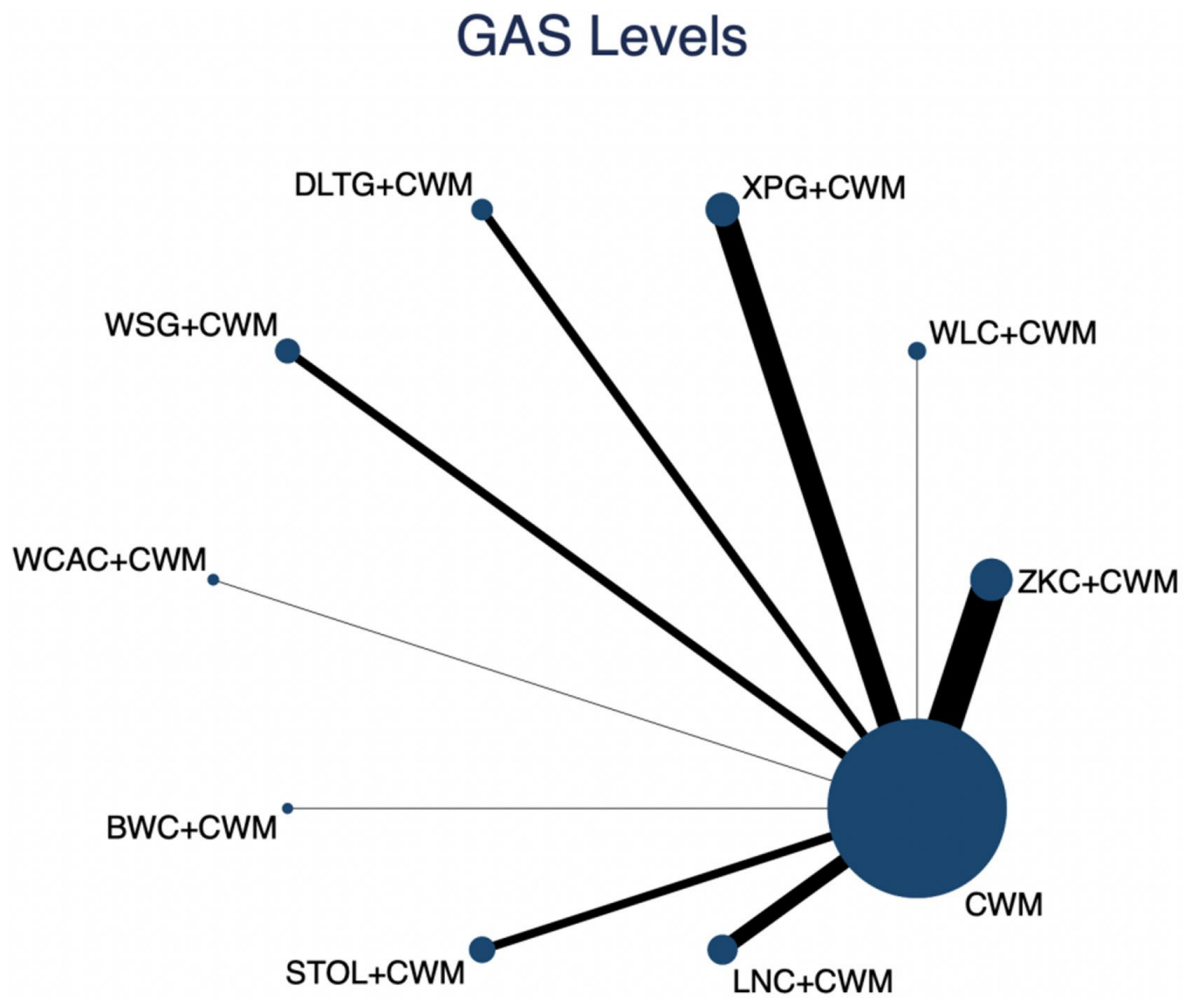
Network of available comparisons for gastrin (GAS) levels. The size of each node is proportional to the total number of randomized participants allocated to that intervention, and the thickness of the connecting lines reflects the number of participants included in head-to-head trials. Interventions include CWM alone and CWM combined with one of twelve CPMs: Zhizhu Kuanzhong Capsules (ZKC), Wuling Capsules (WLC), Qizhi Weitong Granules (QWG), Xiangsha Pingwei Granules (XPG), Dalitong Granules (DLTG), Weisu Granules (WSG), Jinghua Weikang Capsules (JWC), Weichang An Capsules (WCAC), Bilin Weitong Granules (BWC), Simo Tang Oral Liquid (STOL), Liuwei Nengxiao Capsules (LNC), and Liuwei Anxiao Capsules (LAC).

**Figure 10:**
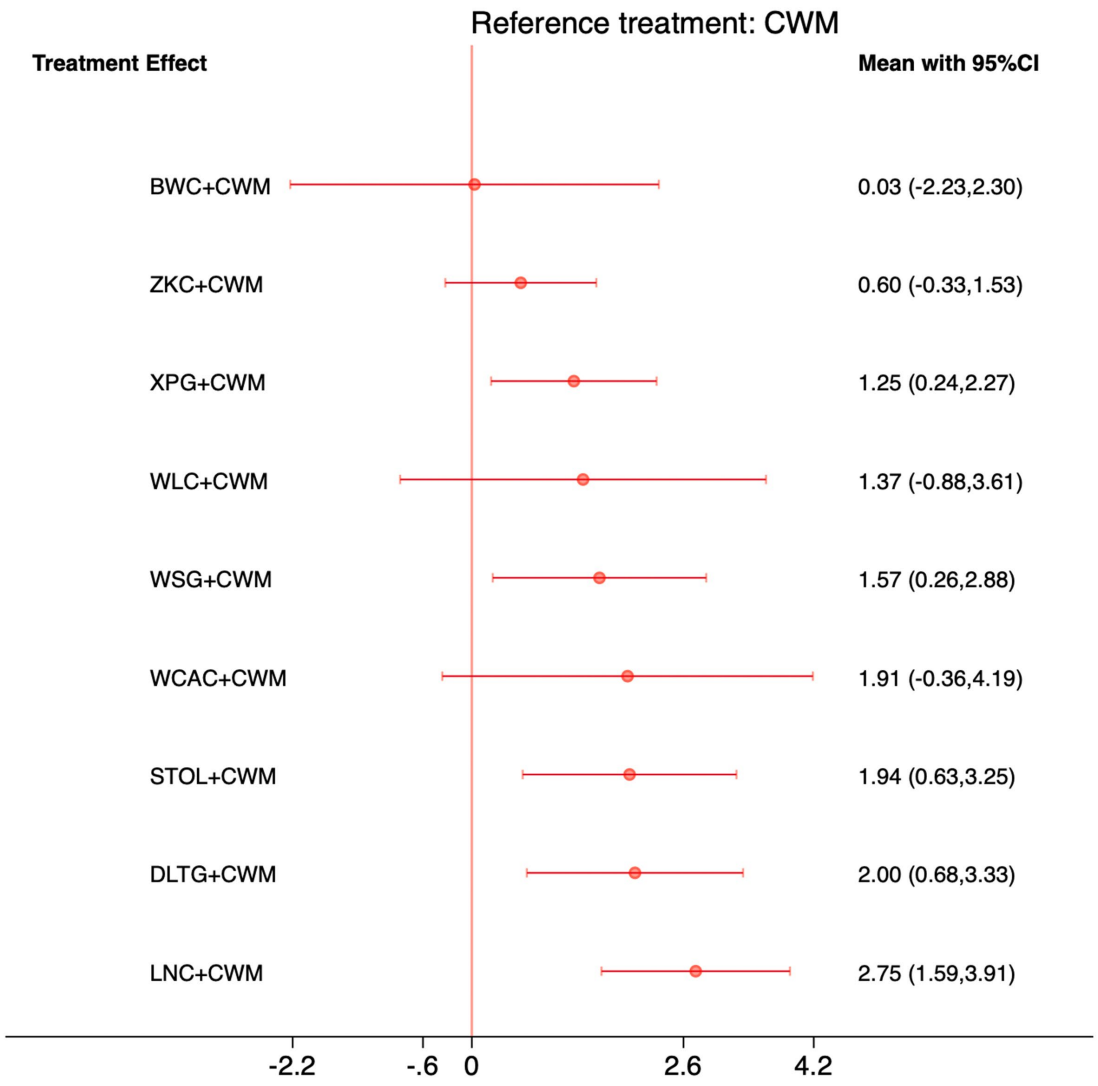
Forest plot of network meta-analysis comparing CPMs combined with CWM versus CWM alone for gastrin (GAS) levels. Effect sizes are expressed as mean differences (MDs) with 95% confidence intervals, using CWM as the common reference treatment. Values to the right of the vertical line indicate higher GAS levels compared with CWM.

**Figure 11:**
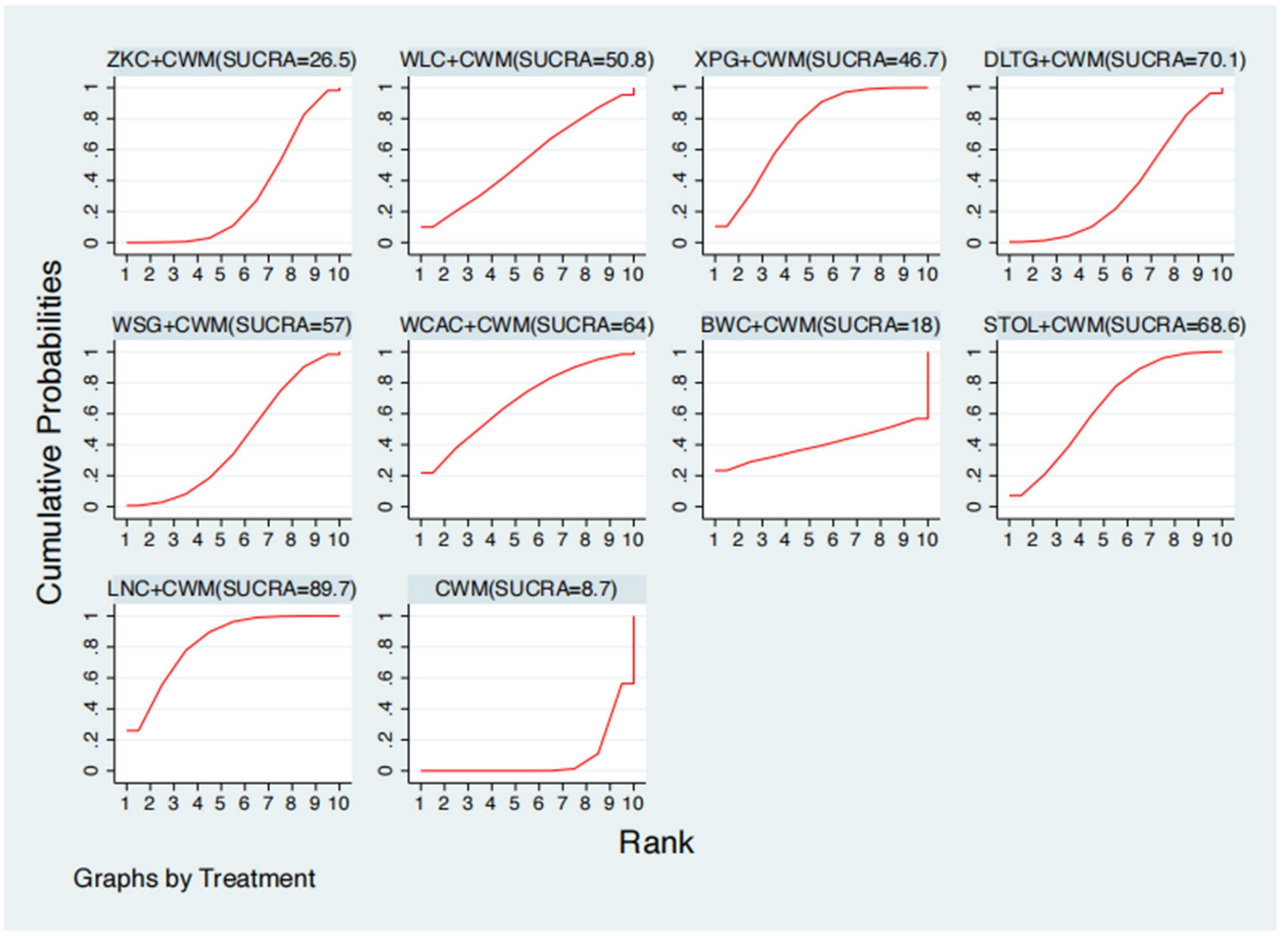
Surface under the cumulative ranking curve (SUCRA) for gastrin (GAS) levels.

This network meta-analysis included 27 RCTs involving 2,684 participants who reported data on GAS levels. The network plot demonstrated that all 9 CPMs combined with CWM were compared, directly or indirectly, with CWM alone (Figure 9). Node size was proportional to the number of participants in each intervention, and line thickness reflected the number of trials directly comparing two treatments. Overall, the forest plot indicated that several CPM + CWM regimens were associated with significantly increased GAS levels compared with CWM alone (Figure 10). The most substantial improvements were observed with LNC + CWM (MD = 2.75; 95% CI 1.59–3.91; SUCRA = 89.7%), followed by DLTG + CWM (MD = 2.00; 95% CI 0.68–3.33; SUCRA = 70.1%) and STOL + CWM (MD = 1.94; 95% CI 0.63–3.25; SUCRA = 68.6%) (Figure 11). Indirect comparisons among CPM regimens based on the league table revealed that LNC + CWM was significantly superior to both ZKC + CWM (MD = −2.15; 95% CI −3.63 to −0.67) and BWC + CWM (MD = −2.72; 95% CI −5.26 to −0.17). The complete league table of relative effect estimates is presented in Appendix 6 (Table S3).

### Adverse event rates

**Figure 12:**
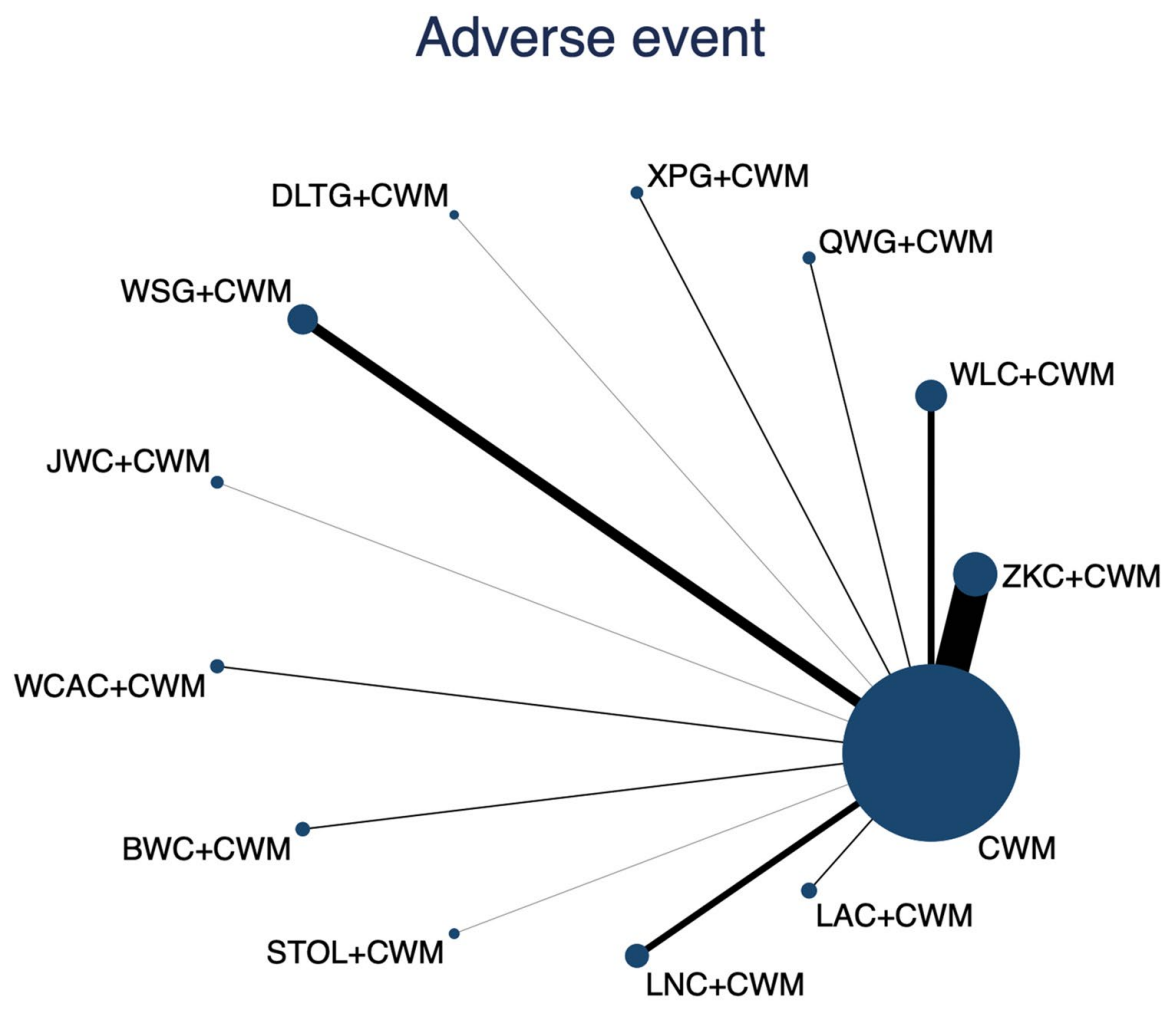
Network of available comparisons for adverse events. The size of each node is proportional to the total number of randomized participants allocated to that intervention, and the thickness of the connecting lines reflects the number of participants included in head-to-head trials. Interventions include CWM alone and CWM combined with one of twelve CPMs: Zhizhu Kuanzhong Capsules (ZKC), Wuling Capsules (WLC), Qizhi Weitong Granules (QWG), Xiangsha Pingwei Granules (XPG), Dalitong Granules (DLTG), Weisu Granules (WSG), Jinghua Weikang Capsules (JWC), Weichang An Capsules (WCAC), Bilin Weitong Granules (BWC), Simo Tang Oral Liquid (STOL), Liuwei Nengxiao Capsules (LNC), and Liuwei Anxiao Capsules (LAC).

**Figure 13:**
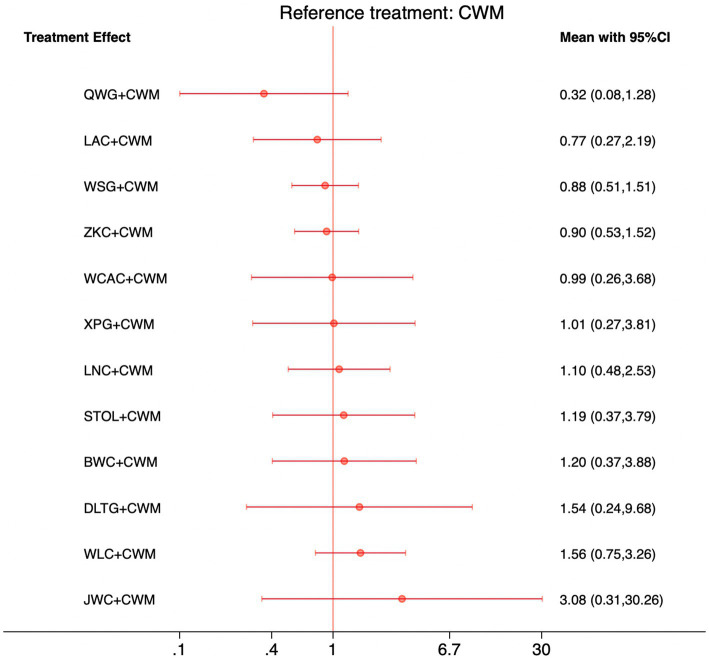
Forest plot of network meta-analysis comparing CPMs combined with CWM versus CWM alone for adverse events. Effect sizes are expressed as odds ratios (ORs) with 95% confidence intervals, using CWM as the common reference treatment. Values to the left of the vertical line indicate a lower probability of adverse events compared with CWM, while values to the right indicate a higher probability.

This network meta-analysis included 35 RCTs involving 3,516 participants who reported data on adverse events. The network plot demonstrated that all 12 CPMs combined with CWM were compared, directly or indirectly, with CWM alone (Figure 12). Node size was proportional to the number of participants in each intervention, and line thickness reflected the number of trials directly comparing two treatments. Overall, the forest plot indicated that none of the CPM + CWM regimens were associated with statistically significant differences in adverse event rates compared with CWM alone (Figure 13). Indirect comparisons based on the league table showed only one significant difference: WLC + CWM was associated with a higher risk of adverse events compared with QWG + CWM (OR = 4.82; 95% CI 1.02–22.87). Regarding safety rankings, SUCRA values suggested that QWG + CWM (91.5%), LAC + CWM (64.8%), and ZKC + CWM (59.3%) were among the most favorable in terms of tolerability, whereas JWC + CWM (19.9%) and WLC + CWM (27.6%) ranked lower. However, given the wide confidence intervals and the absence of consistent statistically significant differences, these rankings should be interpreted with caution. The complete league table of relative effect estimates is presented in Appendix 6 (Table S4), and the SUCRA rankings are provided in Appendix 5 (Figure S1).

### Sensitivity analyses and meta-regression

To examine the robustness of our findings, we performed sensitivity analyses for all primary outcomes (Appendix 9). First, we excluded studies judged to be at high risk of bias; the results were consistent with the main analyses, indicating that study quality did not substantially influence the conclusions. Second, we excluded small-sample studies (<30 participants per arm). In addition, meta-regression analyses were conducted to assess the potential impact of baseline covariates, including age, disease duration, and treatment duration. Across all outcomes, the 95% confidence intervals for these covariates crossed the null, suggesting no significant effect modification. Together, these analyses support the stability and reliability of our results (Appendix 10).

## Discussion

### Primary findings

This network meta-analysis comprehensively evaluated the comparative efficacy and safety of 12 CPMs combined with CWM in the treatment of FD. Across 76 RCTs, four clinically relevant outcomes were assessed: total effective rate, serum MTL levels, serum GAS levels, and adverse event rates. Overall, CPM + CWM regimens were consistently associated with higher clinical effectiveness and improvements in gastrointestinal hormone levels compared with CWM alone, while safety profiles remained comparable. Among the evaluated interventions, JWC + CWM demonstrated the most robust benefits for overall symptom improvement. WLC + CWM were most effective in enhancing MTL levels, whereas LNC + CWM showed superiority in improving GAS levels. With regard to safety, no CPM regimen was associated with a significantly increased risk of adverse events compared with CWM, though some rankings suggested potential differences in tolerability. Taken together, these findings indicate that the addition of CPMs to standard Western therapies may offer clinically meaningful improvements for patients with FD, while maintaining acceptable safety profiles.

### Comparison with previous studies

The global incidence of functional dyspepsia has shown an upward trend in recent years, with affected individuals frequently experiencing psychological comorbidities. However, whether these comorbidities precede or follow FD symptoms remains unclear (97). Current treatment strategies remain largely symptomatic, reflecting the limited understanding of FD pathophysiology. For example, abdominal pain and motility disorders are characteristic symptoms of FD, but commonly used analgesics and central nervous system modulators may alleviate pain while exacerbating gastrointestinal motility problems. This therapeutic dilemma underscores the need for more effective and targeted approaches.

Accumulating evidence from randomized controlled trials and systematic reviews indicates that combining CPM with CWM is more effective than CWM alone (98). Nevertheless, clinical practice has lacked robust comparative data across different CPM formulations. Our study addresses this gap by providing head-to-head and indirect evidence on their relative efficacy and safety.

Motilin plays a key role in gastrointestinal motility regulation, closely linked to migrating motor complexes (MMCs), which cyclically clear the intestine of debris and bacteria during fasting. MTL secretion peaks prior to the most vigorous phase of peristalsis (Phase III) and also promotes pepsin release and gastric emptying (99). In our analysis, WLC + CWM, ZKC + CWM, and STOL + CWM demonstrated the greatest improvements in serum MTL levels, highlighting their ability to enhance gastrointestinal motility. These findings are consistent with pharmacological evidence that herbal compounds can modulate motility, visceral sensitivity, and the gut-brain axis.

Similarly, gastrin is a hormone essential for stimulating gastric acid secretion and motility. Beyond increasing acid secretion, GAS enhances antral contractions, delays gastric emptying, promotes pepsin and bile secretion, and augments overall gastrointestinal activity. Our study found that LNC + CWM ranked highest in improving serum GAS levels, followed by DLTG + CWM and STOL + CWM. Liuwei Nengxiao Capsules (LNC), a widely used and cost-effective CPM, contain multiple components such as *Rheum officinale*, *Zingiber officinale*, Saussurea lappa, and Calcii Sulfas Exsiccatus. Modern pharmacological studies suggest these act synergistically to regulate lipids, protect the gastric mucosa, stimulate peristalsis, and neutralize gastric acid (100). These mechanisms likely underlie the observed improvements in GAS levels and clinical outcomes. By integrating clinical trial evidence with mechanistic insights, our findings reinforce the therapeutic potential of CPMs in modulating key gastrointestinal hormones and improving symptom control in FD.

### Discussion on syndrome differentiation and individualized treatment

TCM emphasizes individualized treatment based on syndrome differentiation, a principle that may help explain the varying efficacy observed among different CPMs when combined with CWM for functional dyspepsia. From a TCM perspective, functional dyspepsia is a multifactorial disorder involving several common syndromes, including liver–stomach disharmony, spleen–stomach deficiency, and damp-heat accumulation. Each CPM is formulated to address specific pathophysiological patterns and symptom profiles within these syndromes.

For instance, JWC is designed to harmonize the liver and stomach and relieve stagnation, which may explain their pronounced benefits in overall symptom improvement among patients with liver–stomach disharmony. WLC focuses on strengthening the spleen and invigorating the stomach, aligning with spleen–stomach deficiency syndromes and consistent with their observed effect on MTL levels. LNC possesses heat-clearing and qi-regulating properties, potentially benefiting patients with damp-heat or excess syndromes, consistent with their superior performance in regulating GAS levels.

Together, these findings suggest that the clinical efficacy of CPMs depends partly on how well their therapeutic actions correspond to underlying syndrome patterns. Incorporating principles of syndrome differentiation and individualized treatment into future clinical research and trial designs may improve therapeutic precision and enhance the real-world relevance of TCM studies on functional dyspepsia.

### Limitation

This study utilized a network meta-analysis to compare the efficacy of different traditional Chinese medicines combined with conventional Western medicine in the treatment of FD. However, there are several limitations to this study: (1) The number of studies included for specific interventions was relatively small, leading to less precise comparison results, as indicated by 95% confidence intervals (CI) that included or approached the null effect. (2) Due to limitations in the original data, the range of outcome measures included in this study was somewhat restricted, preventing a comprehensive assessment of more clinical indicators, and lacking follow-up data to reflect the long-term efficacy of TCMs. (3) The comparisons between TCMs were indirect. (4) Although the search covered both Chinese and English databases, most of the included studies were published in Chinese, and the proportion of international English-language studies was relatively low, which may introduce potential regional or publication bias. Future studies should aim to include more international literature to improve the generalizability of the findings. (5) The “total effective rate” used as a primary outcome in many included trials is a semi-subjective composite indicator without a universally accepted definition. Variations in the evaluation criteria across studies may introduce inconsistency and potential measurement bias. Therefore, the interpretation of this outcome should be cautious, and future research should adopt more objective and standardized endpoints (e.g., validated symptom scales or quality-of-life measures) to enhance comparability and reliability. In summary, these limitations suggest that while our findings provide a valuable overview of current evidence, they should be interpreted with caution until further high-quality, internationally representative, and methodologically standardized studies become available.

### Clinical implications

This study provides comprehensive comparative evidence on the efficacy and safety of 12 CPMs combined with CWM for FD. The results indicate that CPM–CWM combinations generally outperform CWM alone in improving clinical symptoms and regulating gastrointestinal hormones, without increasing adverse events. These findings suggest that integrating CPMs into standard treatment may provide a feasible and safe complementary strategy for managing FD. From a clinical perspective, this evidence supports a more nuanced approach to patient care. For instance, JWC may be prioritized for patients with prominent postprandial discomfort or epigastric distention, WLC for those with motility-related dysfunction reflected by low motilin levels, and LNC for those with excessive gastric acid secretion. Such differentiation aligns with TCM principles of individualized therapy and may help bridge the gap between traditional pattern-based prescribing and modern evidence-based medicine.

Notably, the comparable safety profiles across interventions underscore the clinical feasibility of integrating CPMs as adjunctive therapies rather than replacements for standard care. Nevertheless, individualized treatment remains essential—both to match therapeutic mechanisms with symptom clusters and to minimize unnecessary medication use. Future clinical trials that stratify patients by syndrome type and employ standardized outcome measures will be critical for translating these findings into optimized, evidence-informed treatment protocols.

## Conclusion

This network meta-analysis provides the most comprehensive synthesis to date on the efficacy and safety of CPMs combined with CWM for functional dyspepsia. Overall, CPM + CWM regimens were consistently more effective than CWM alone in improving total symptom response, motilin, and gastrin levels, while showing comparable safety profiles. Importantly, no substantial increase in adverse events was observed, supporting the tolerability of these integrative strategies. Despite limitations in study quality and reporting, our findings highlight the clinical potential of CPMs as valuable adjuncts to standard pharmacotherapy. Future large-scale, rigorously designed RCTs with standardized outcome definitions are warranted to strengthen the evidence base and clarify the role of specific CPMs in personalized management of functional dyspepsia.

## Data Availability

The original contributions presented in the study are included in the article/supplementary material, further inquiries can be directed to the corresponding author.
