# Supplementary material for: Twelve Chinese patent medicines combined with conventional medicine for the treatment of functional dyspepsia: a network meta-analysis
Source: Front Med (Lausanne). 2025 Dec 12;12:1670153. doi: 10.3389/fmed.2025.1670153 (PMC12741128; doi:10.3389/fmed.2025.1670153)
Supplement: Supplementary file 1 [file Data_Sheet_1.docx]

**Appendix 1: PRISMA NMA Checklist**

| **Section/Topic** | **Item #** | **Checklist Item** | **Reported on Page #** |
| --- | --- | --- | --- |
| **TITLE** |  |  |  |
| Title | 1 | Identify the report as a systematic review *incorporating a network meta-analysis (or related form of meta-analysis).* | 1 |
|  |  |  |  |
| **ABSTRACT** |  |  |  |
| Structured summary | 2 | Provide a structured summary including, as applicable:  **Background:** main objectives  **Methods:** data sources; study eligibility criteria, participants, and interventions; study appraisal; and *synthesis methods, such as network meta-analysis.*  **Results:** number of studies and participants identified; summary estimates with corresponding confidence/credible intervals; *treatment rankings may also be discussed. Authors may choose to summarize pairwise comparisons against a chosen treatment included in their analyses for brevity.*  **Discussion/Conclusions:** limitations; conclusions and implications of findings.  **Other:** primary source of funding; systematic review registration number with registry name. | 2 |
|  |  |  |  |
| **INTRODUCTION** |  |  |  |
| Rationale | 3 | Describe the rationale for the review in the context of what is already known*, including mention of why a network meta-analysis has been conducted.* | ***2*** |
| Objectives | 4 | Provide an explicit statement of questions being addressed, with reference to participants, interventions, comparisons, outcomes, and study design (PICOS). | 3 |
|  |  |  |  |
| **METHODS** |  |  |  |
| Protocol and registration | 5 | Indicate whether a review protocol exists and if and where it can be accessed (e.g., Web address); and, if available, provide registration information, including registration number. | 3 |
| Eligibility criteria | 6 | Specify study characteristics (e.g., PICOS, length of follow-up) and report characteristics (e.g., years considered, language, publication status) used as criteria for eligibility, giving rationale. *Clearly describe eligible treatments included in the treatment network, and note whether any have been clustered or merged into the same node (with justification).* | ***3*** |
| Information sources | 7 | Describe all information sources (e.g., databases with dates of coverage, contact with study authors to identify additional studies) in the search and date last searched. | 3 |
| Search | 8 | Present full electronic search strategy for at least one database, including any limits used, such that it could be repeated. | 3&**Appendix 2** |
| Study selection | 9 | State the process for selecting studies (i.e., screening, eligibility, included in systematic review, and, if applicable, included in the meta-analysis). | 4 |
| Data collection process | 10 | Describe method of data extraction from reports (e.g., piloted forms, independently, in duplicate) and any processes for obtaining and confirming data from investigators. | 4 |
| Data items | 11 | List and define all variables for which data were sought (e.g., PICOS, funding sources) and any assumptions and simplifications made. | 4 |
| **Geometry of the network** | **S1** | Describe methods used to explore the geometry of the treatment network under study and potential biases related to it. This should include how the evidence base has been graphically summarized for presentation, and what characteristics were compiled and used to describe the evidence base to readers. | ***5*** |
| Risk of bias within individual studies | 12 | Describe methods used for assessing risk of bias of individual studies (including specification of whether this was done at the study or outcome level), and how this information is to be used in any data synthesis. | 5 |
| Summary measures | 13 | State the principal summary measures (e.g., risk ratio, difference in means). *Also describe the use of additional summary measures assessed, such as treatment rankings and surface under the cumulative ranking curve (SUCRA) values, as well as modified approaches used to present summary findings from meta-analyses.* | 5 |
| Planned methods of analysis | 14 | Describe the methods of handling data and combining results of studies for each network meta-analysis. This should include, but not be limited to:   - *Handling of multi-arm trials;* - *Selection of variance structure;* - *Selection of prior distributions in Bayesian analyses; and* - *Assessment of model fit.* | 5 |
| **Assessment of Inconsistency** | **S2** | Describe the statistical methods used to evaluate the agreement of direct and indirect evidence in the treatment network(s) studied. Describe efforts taken to address its presence when found. | 5 |
| Risk of bias across studies | 15 | Specify any assessment of risk of bias that may affect the cumulative evidence (e.g., publication bias, selective reporting within studies). | **5** |
| Additional analyses | 16 | Describe methods of additional analyses if done, indicating which were pre-specified. This may include, but not be limited to, the following:   - Sensitivity or subgroup analyses; - Meta-regression analyses; - *Alternative formulations of the treatment network; and* - *Use of alternative prior distributions for Bayesian analyses (if applicable).* | ***5*** |
| **RESULTS†** |  |  |  |
| Study selection | 17 | Give numbers of studies screened, assessed for eligibility, and included in the review, with reasons for exclusions at each stage, ideally with a flow diagram. | 6 |
| **Presentation of network structure** | **S3** | Provide a network graph of the included studies to enable visualization of the geometry of the treatment network. | ***10*** |
| **Summary of network geometry** | **S4** | Provide a brief overview of characteristics of the treatment network. This may include commentary on the abundance of trials and randomized patients for the different interventions and pairwise comparisons in the network, gaps of evidence in the treatment network, and potential biases reflected by the network structure. | ***10*** |
| Study characteristics | 18 | For each study, present characteristics for which data were extracted (e.g., study size, PICOS, follow-up period) and provide the citations. | 7 |
| Risk of bias within studies | 19 | Present data on risk of bias of each study and, if available, any outcome level assessment. | 8 |
| Results of individual studies | 20 | For all outcomes considered (benefits or harms), present, for each study: 1) simple summary data for each intervention group, and 2) effect estimates and confidence intervals. *Modified approaches may be needed to deal with information from larger networks.* | ***Appendix*** |
| Synthesis of results | 21 | Present results of each meta-analysis done, including confidence/credible intervals. *In larger networks, authors may focus on comparisons versus a particular comparator (e.g. placebo or standard care), with full findings presented in an appendix. League tables and forest plots may be considered to summarize pairwise comparisons.* If additional summary measures were explored (such as treatment rankings), these should also be presented. | 9 |
| **Exploration for inconsistency** | **S5** | Describe results from investigations of inconsistency. This may include such information as measures of model fit to compare consistency and inconsistency models, *P* values from statistical tests, or summary of inconsistency estimates from different parts of the treatment network. | ***9*** |
| Risk of bias across studies | 22 | Present results of any assessment of risk of bias across studies for the evidence base being studied. | ***Appendix*** |
| Results of additional analyses | 23 | Give results of additional analyses, if done (e.g., sensitivity or subgroup analyses, meta-regression analyses*, alternative network geometries studied, alternative choice of prior distributions for Bayesian analyses,* and so forth). | ***none*** |
| **DISCUSSION** |  |  |  |
| Summary of evidence | 24 | Summarize the main findings, including the strength of evidence for each main outcome; consider their relevance to key groups (e.g., healthcare providers, users, and policy-makers). | 17 |
| Limitations | 25 | Discuss limitations at study and outcome level (e.g., risk of bias), and at review level (e.g., incomplete retrieval of identified research, reporting bias). *Comment on the validity of the assumptions, such as transitivity and consistency. Comment on any concerns regarding network geometry (e.g., avoidance of certain comparisons).* | 19 |
| Conclusions | 26 | Provide a general interpretation of the results in the context of other evidence, and implications for future research. | 20 |
| **FUNDING** |  |  |  |
| Funding | 27 | Describe sources of funding for the systematic review and other support (e.g., supply of data); role of funders for the systematic review. This should also include information regarding whether funding has been received from manufacturers of treatments in the network and/or whether some of the authors are content experts with professional conflicts of interest that could affect use of treatments in the network. | ***Title page*** |

**Appendix 2: Search strategy**

**Table S1.** Search strategy of PubMed

| **#** | **Searches** |
| --- | --- |
| 1 | ((((“Functional Dyspepsia”[MeSH Terms]) OR “Functional Dyspepsia”[Title/Abstract]) OR “FD”[Title/Abstract]) |
| 2 | ((“Zhizhu Kuanzhong Capsules”[Title/Abstract] OR “ZKC”[Title/Abstract]) OR (“Wuling Capsules”[Title/Abstract] OR “WLC”[Title/Abstract]) OR (“Qizhi Weitong Granules”[Title/Abstract] OR “QWG”[Title/Abstract]) OR (“Xiangsha Pingwei Granules”[Title/Abstract] OR “XPG”[Title/Abstract]) OR (“Dalitong Granules”[Title/Abstract] OR “DLTG”[Title/Abstract]) OR (“Weisu Granules”[Title/Abstract] OR “WSG”[Title/Abstract]) OR (“Jinghua Weikang Capsules”[Title/Abstract] OR “JWC”[Title/Abstract]) OR (“Weichang An Capsules”[Title/Abstract] OR “WCAC”[Title/Abstract]) OR (“Bilin Weitong Granules”[Title/Abstract] OR “BWC”[Title/Abstract]) OR (“Simo Tang Oral Liquid”[Title/Abstract] OR “STOL”[Title/Abstract]) OR (“Liuwei Nengxiao Capsules”[Title/Abstract] OR “LNC”[Title/Abstract]) OR (“Liuwei Anxiao Capsules”[Title/Abstract] OR “LAC”[Title/Abstract])) |
| 3 | (“Randomized Controlled Trial”[Publication Type] OR “Randomized Controlled Trials as Topic”[MeSH Terms] OR randomized[Title/Abstract] OR randomly[Title/Abstract] OR RCT[Title/Abstract]) |
| 4 | #1 AND #2 AND #3 |

**Table S2.** Search strategy of Web of Science

| **#** | **Searches** |
| --- | --- |
| 1 | TS=(“Functional Dyspepsia” OR “FD”) |
| 2 | TS=(“Zhizhu Kuanzhong Capsules” OR “ZKC” OR “Wuling Capsules” OR “WLC” OR “Qizhi Weitong Granules” OR “QWG” OR “Xiangsha Pingwei Granules” OR “XPG” OR “Dalitong Granules” OR “DLTG” OR “Weisu Granules” OR “WSG” OR “Jinghua Weikang Capsules” OR “JWC” OR “Weichang An Capsules” OR “WCAC” OR “Bilin Weitong Granules” OR “BWC” OR “Simo Tang Oral Liquid” OR “STOL” OR “Liuwei Nengxiao Capsules” OR “LNC” OR “Liuwei Anxiao Capsules” OR “LAC”) |
| 3 | TS=("Randomized Controlled Trial" OR "Randomized Controlled Trials as Topic" OR randomized OR randomly OR RCT) |
| 4 | #1 AND #2 AND #3 |

**Table S3.** Search strategy of Cochrane Central Register of Controlled Trials

| **#** | **Searches** |
| --- | --- |
| 1 | (“Functional Dyspepsia” OR FD):ti,ab,kw |
| 2 | (“Zhizhu Kuanzhong Capsules” OR ZKC OR “Wuling Capsules” OR WLC OR “Qizhi Weitong Granules” OR QWG OR “Xiangsha Pingwei Granules” OR XPG OR “Dalitong Granules” OR DLTG OR “Weisu Granules” OR WSG OR “Jinghua Weikang Capsules” OR JWC OR “Weichang An Capsules” OR WCAC OR “Bilin Weitong Granules” OR BWC OR “Simo Tang Oral Liquid” OR STOL OR “Liuwei Nengxiao Capsules” OR LNC OR “Liuwei Anxiao Capsules” OR LAC):ti,ab,kw |
| 3 | (“randomized controlled trial” OR RCT OR randomized OR randomly):ti,ab,kw |
| 4 | #1 AND #2 AND #3 |

**Table S4.** Search strategy of Embase

| **#** | **Searches** |
| --- | --- |
| 1 | (‘functional dyspepsia’/exp OR ‘functional dyspepsia’:ti,ab OR FD:ti,ab) |
| 2 | (‘zhizhu kuanzhong capsules’:ti,ab OR ZKC:ti,ab OR ‘wuling capsules’:ti,ab OR WLC:ti,ab OR ‘qizhi weitong granules’:ti,ab OR QWG:ti,ab OR ‘xiangsha pingwei granules’:ti,ab OR XPG:ti,ab OR ‘dalitong granules’:ti,ab OR DLTG:ti,ab OR ‘weisu granules’:ti,ab OR WSG:ti,ab OR ‘jinghua weikang capsules’:ti,ab OR JWC:ti,ab OR ‘weichang an capsules’:ti,ab OR WCAC:ti,ab OR ‘bilin weitong granules’:ti,ab OR BWC:ti,ab OR ‘simo tang oral liquid’:ti,ab OR STOL:ti,ab OR ‘liu wei nengxiao capsules’:ti,ab OR LNC:ti,ab OR ‘liu wei anxiao capsules’:ti,ab OR LAC:ti,ab) |
| 3 | (‘randomized controlled trial’/exp OR randomized:ti,ab OR randomly:ti,ab OR RCT:ti,ab) |
| 4 | #1 AND #2 AND #3 |

**Table S5.** Search strategy of CNKI, VIP, Wanfang, Sinomed

| **#** | **Searches** |
| --- | --- |
| 1 | “功能性消化不良” OR “FD” |
| 2 | “枳术宽中胶囊” OR “ZKC” OR “乌灵胶囊” OR “WLC” OR “气滞胃痛颗粒” OR “QWG” OR “香砂平胃颗粒” OR “XPG” OR “达立通颗粒” OR “DLTG” OR “胃苏颗粒” OR “WSG” OR “荆花胃康胶丸” OR “JWC” OR “胃肠安胶囊” OR “WCAC” OR “荜铃胃痛颗粒” OR “BWC” OR “四磨汤口服液” OR “STOL” OR “六味能消胶囊” OR “LNC” OR “六味安消胶囊” OR “LAC” |
| 3 | “随机对照试验” OR “随机” OR “对照试验” OR “RCT” |
| 4 | #1 AND #2 AND #3 |

**Appendix 3: Risk of bias of randomized clinical trials.**


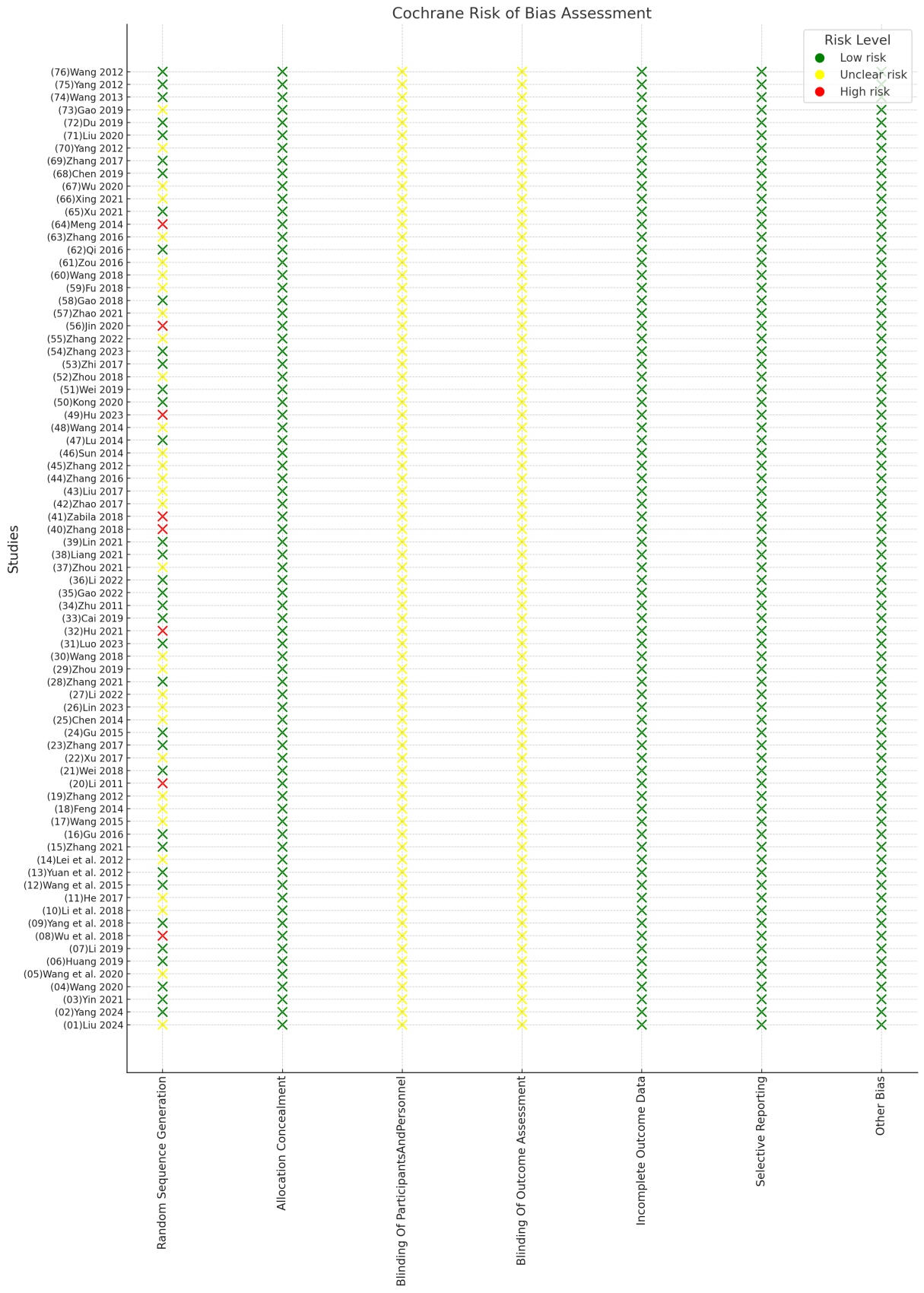


**Appendix 4: Evaluation of inconsistency and heterogeneity**

**Table S4.1:** Global consistency

| **Outcome** | **Consistency** | | **Heterogeneity**  （τ^2^） |
| --- | --- | --- | --- |
|  | Chi^2^ | *P* |  |
| Total effective rate | 2.18 | 0.1399 | 0.0157034 |
| MTL Levels | 0.34 | 0.5599 | 0.181276 |
| GAS Levels | 1.09 | 0.2972 | 0.447837 |
| Adverse Event Rates | 4.00 | 0.4555 | 0.116376 |

**Appendix 5. SUCRA plots of network meta-analysis**

**Figure S1. Surface under the cumulative ranking curve (SUCRA) for adverse events**

**
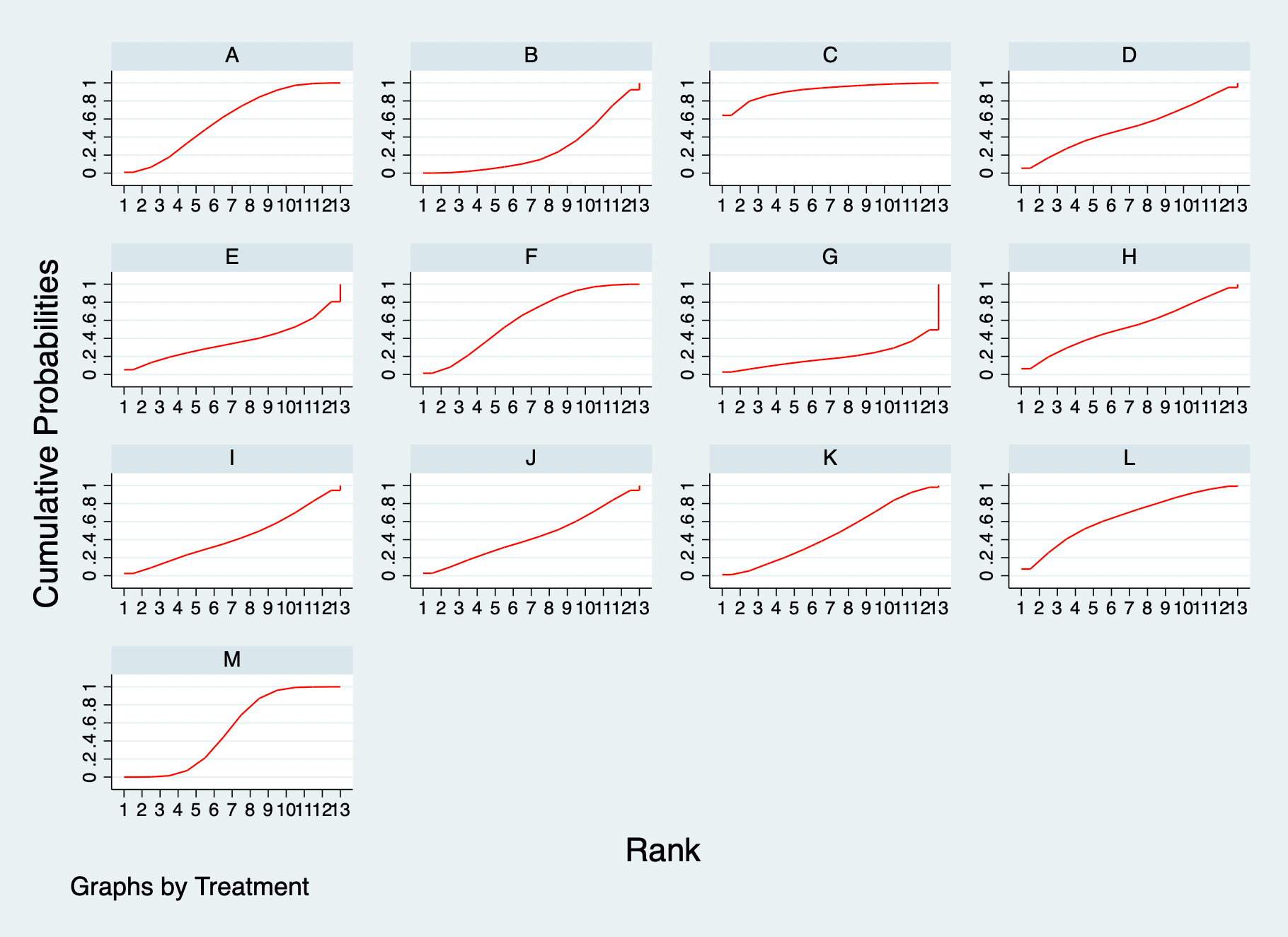
**

Note: A = ZKC + CWM; B = WLC + CWM; C = QWG + CWM; D = XPG + CWM; E = DLTG + CWM; F = WSG + CWM; G = JWC + CWM; H = WCAC + CWM; I = BWC + CWM; J = STOL + CWM; K = LNC + CWM; L = LAC + CWM; M = CWM

| Treatment | SUCRA |
| --- | --- |
| ZKC+CWM | 59.3 |
| WLC+CWM | 27.6 |
| QWG+CWM | 91.5 |
| XPG+CWM | 51 |
| DLTG+CWM | 36.6 |
| WSG+CWM | 59.1 |
| JWC+CWM | 19.9 |
| WCAC+CWM | 53.6 |
| BWC+CWM | 42.7 |
| STOL+CWM | 44.4 |
| LNC+CWM | 46.5 |
| LAC+CWM | 64.8 |
| CWM | 53 |

**Appendix 6. League tables of relative treatment effects**

Table S1. League table of relative treatment effects (risk ratios, 95% CIs) for total effective rate.

| ZKC+CWM |  |  |  |  |  |  |  |  |  |  |  |  |
| --- | --- | --- | --- | --- | --- | --- | --- | --- | --- | --- | --- | --- |
| 0.92 (0.83,1.02) | WLC+CWM |  |  |  |  |  |  |  |  |  |  |  |
| 0.95 (0.85,1.06) | 1.03 (0.90,1.18) | QWG+CWM |  |  |  |  |  |  |  |  |  |  |
| 1.08 (0.97,1.20) | **1.17 (1.03,1.33)** | 1.14 (0.99,1.30) | XPG+CWM |  |  |  |  |  |  |  |  |  |
| 0.97 (0.86,1.08) | 1.05 (0.92,1.20) | 1.02 (0.88,1.17) | 0.89 (0.78,1.03) | DLTG+CWM |  |  |  |  |  |  |  |  |
| 1.00 (0.92,1.07) | 1.08 (0.97,1.20) | 1.05 (0.94,1.17) | 0.92 (0.83,1.03) | 1.03 (0.92,1.16) | WSG+CWM |  |  |  |  |  |  |  |
| 0.82 (0.71,0.94) | 0.89 (0.75,1.04) | 0.86 (0.73,1.01) | **0.76 (0.64,0.89)** | 0.85 (0.72,1.00) | 0.82 (0.71,0.95) | JWC+CWM |  |  |  |  |  |  |
| 0.99 (0.90,1.09) | 1.07 (0.95,1.21) | 1.04 (0.91,1.18) | 0.91 (0.80,1.04) | 1.02 (0.90,1.17) | 0.99 (0.90,1.10) | **1.21 (1.03,1.41)** | WCAC+CWM |  |  |  |  |  |
| 0.99 (0.88,1.12) | 1.08 (0.94,1.24) | 1.04 (0.90,1.21) | 0.92 (0.80,1.06) | 1.03 (0.89,1.19) | 1.00 (0.88,1.13) | **1.21 (1.02,1.44)** | 1.01 (0.88,1.15) | BWC+CWM |  |  |  |  |
| 1.01 (0.93,1.10) | 1.09 (0.98,1.22) | 1.06 (0.94,1.20) | 0.94 (0.83,1.05) | 1.05 (0.93,1.18) | 1.01 (0.93,1.11) | **1.24 (1.06,1.43)** | 1.02 (0.92,1.14) | 1.02 (0.90,1.15) | STOL+CWM |  |  |  |
| 0.99 (0.90,1.09) | 1.08 (0.95,1.21) | 1.04 (0.92,1.19) | 0.92 (0.81,1.04) | 1.03 (0.90,1.17) | 1.00 (0.90,1.10) | **1.21 (1.04,1.42)** | 1.00 (0.89,1.13) | 1.00 (0.87,1.14) | 0.98 (0.89,1.09) | LNC+CWM |  |  |
| 0.98 (0.89,1.09) | 1.07 (0.94,1.21) | 1.04 (0.91,1.18) | 0.91 (0.80,1.03) | 1.02 (0.89,1.16) | 0.99 (0.89,1.09) | **1.20 (1.03,1.41)** | 1.00 (0.89,1.12) | 0.99 (0.87,1.14) | 0.97 (0.88,1.08) | 0.99 (0.88,1.12) | LAC+CWM |  |
| **1.20 (1.13,1.26)** | **1.29 (1.18,1.42)** | **1.26 (1.14,1.38)** | **1.11 (1.01,1.22)** | **1.24 (1.12,1.37)** | **1.20 (1.13,1.27)** | **1.46 (1.28,1.67)** | **1.21 (1.11,1.32)** | **1.20 (1.08,1.34)** | **1.18 (1.11,1.26)** | **1.20 (1.11,1.31)** | **1.21 (1.12,1.32)** | CWM |

Table S2. League table of relative treatment effects (mean differences, 95% CIs) for motilin (MTL) levels.

| ZKC+CWM |  |  |  |  |  |  |  |  |  |  |  |
| --- | --- | --- | --- | --- | --- | --- | --- | --- | --- | --- | --- |
| -0.23 (-3.20,2.74) | WLC+CWM |  |  |  |  |  |  |  |  |  |  |
| 2.27 (-0.69,5.23) | 2.50 (-1.32,6.32) | QWG+CWM |  |  |  |  |  |  |  |  |  |
| 1.73 (-0.37,3.82) | 1.96 (-1.24,5.16) | -0.55 (-3.74,2.64) | XPG+CWM |  |  |  |  |  |  |  |  |
| 0.72 (-1.80,3.24) | 0.95 (-2.54,4.45) | -1.55 (-5.04,1.93) | -1.00 (-3.79,1.79) | DLTG+CWM |  |  |  |  |  |  |  |
| 1.16 (-1.35,3.67) | 1.39 (-2.10,4.88) | -1.11 (-4.59,2.36) | -0.57 (-3.35,2.21) | 0.44 (-2.68,3.55) | WSG+CWM |  |  |  |  |  |  |
| 1.17 (-1.34,3.69) | 1.40 (-2.08,4.89) | -1.10 (-4.58,2.38) | -0.55 (-3.33,2.23) | 0.45 (-2.66,3.57) | 0.02 (-3.09,3.12) | WCAC+CWM |  |  |  |  |  |
| 1.71 (-2.29,5.70) | 1.94 (-2.73,6.61) | -0.56 (-5.23,4.10) | -0.02 (-4.19,4.15) | 0.99 (-3.41,5.39) | 0.55 (-3.84,4.95) | 0.54 (-3.86,4.93) | BWC+CWM |  |  |  |  |
| 0.55 (-1.71,2.81) | 0.78 (-2.53,4.09) | -1.72 (-5.02,1.58) | -1.18 (-3.73,1.38) | -0.17 (-3.09,2.75) | -0.61 (-3.51,2.30) | -0.62 (-3.53,2.29) | -1.16 (-5.41,3.10) | STOL+CWM |  |  |  |
| 0.82 (-1.44,3.08) | 1.05 (-2.26,4.36) | -1.45 (-4.76,1.85) | -0.91 (-3.46,1.65) | 0.10 (-2.82,3.02) | -0.34 (-3.25,2.57) | -0.35 (-3.27,2.56) | -0.89 (-5.15,3.37) | 0.27 (-2.43,2.96) | LNC+CWM |  |  |
| -0.59 (-4.59,3.41) | -0.36 (-5.03,4.32) | -2.86 (-7.53,1.81) | -2.31 (-6.49,1.86) | -1.31 (-5.72,3.10) | -1.75 (-6.15,2.66) | -1.76 (-6.16,2.64) | -2.30 (-7.68,3.09) | -1.14 (-5.40,3.12) | -1.41 (-5.67,2.86) | LAC+CWM |  |
| **2.62 (1.41,3.84)** | **2.85 (0.15,5.56)** | 0.35 (-2.34,3.05) | 0.90 (-0.81,2.60) | 1.90 (-0.31,4.11) | 1.47 (-0.73,3.66) | 1.45 (-0.75,3.65) | 0.91 (-2.89,4.72) | **2.07 (0.17,3.98)** | 1.80 (-0.10,3.71) | 3.21 (-0.60,7.02) | CWM |

Table S3. League table of relative treatment effects (mean differences, 95% CIs) for gastrin (GAS) levels.

| ZKC+CWM |  |  |  |  |  |  |  |  |  |
| --- | --- | --- | --- | --- | --- | --- | --- | --- | --- |
| -0.76 (-3.19,1.67) | WLC+CWM |  |  |  |  |  |  |  |  |
| -0.65 (-2.03,0.72) | 0.11 (-2.35,2.58) | XPG+CWM |  |  |  |  |  |  |  |
| -1.40 (-3.02,0.22) | -0.64 (-3.25,1.97) | -0.75 (-2.42,0.92) | DLTG+CWM |  |  |  |  |  |  |
| -0.97 (-2.57,0.64) | -0.20 (-2.80,2.40) | -0.31 (-1.97,1.34) | 0.44 (-1.43,2.30) | WSG+CWM |  |  |  |  |  |
| -1.31 (-3.77,1.15) | -0.55 (-3.74,2.65) | -0.66 (-3.15,1.83) | 0.09 (-2.54,2.73) | -0.34 (-2.97,2.28) | WCAC+CWM |  |  |  |  |
| 0.57 (-1.88,3.01) | 1.33 (-1.86,4.52) | 1.22 (-1.26,3.70) | 1.97 (-0.65,4.59) | 1.53 (-1.08,4.15) | 1.88 (-1.33,5.09) | BWC+CWM |  |  |  |
| -1.34 (-2.94,0.27) | -0.57 (-3.17,2.03) | -0.68 (-2.34,0.98) | 0.07 (-1.80,1.93) | -0.37 (-2.22,1.49) | -0.02 (-2.65,2.60) | -1.90 (-4.52,0.71) | STOL+CWM |  |  |
| **-2.15 (-3.63,-0.67)** | -1.38 (-3.91,1.14) | -1.50 (-3.04,0.04) | -0.75 (-2.51,1.01) | -1.18 (-2.93,0.57) | -0.84 (-3.39,1.72) | **-2.72 (-5.26,-0.17)** | -0.81 (-2.56,0.94) | LNC+CWM |  |
| 0.60 (-0.33,1.53) | 1.37 (-0.88,3.61) | **1.25 (0.24,2.27)** | **2.00 (0.68,3.33)** | **1.57 (0.26,2.88)** | 1.91 (-0.36,4.19) | 0.03 (-2.23,2.30) | **1.94 (0.63,3.25)** | **2.75 (1.59,3.91)** | CWM |

Table S4. League table of relative treatment effects (odds ratios, 95% CIs) for adverse events.

| ZKC+CWM |  |  |  |  |  |  |  |  |  |  |  |  |
| --- | --- | --- | --- | --- | --- | --- | --- | --- | --- | --- | --- | --- |
| 0.58 (0.23,1.42) | WLC+CWM |  |  |  |  |  |  |  |  |  |  |  |
| 2.78 (0.64,12.06) | **4.82 (1.02,22.87)** | QWG+CWM |  |  |  |  |  |  |  |  |  |  |
| 0.89 (0.21,3.69) | 1.54 (0.34,7.01) | 0.32 (0.05,2.15) | XPG+CWM |  |  |  |  |  |  |  |  |  |
| 0.59 (0.09,3.97) | 1.02 (0.14,7.38) | 0.21 (0.02,2.09) | 0.66 (0.07,6.37) | DLTG+CWM |  |  |  |  |  |  |  |  |
| 1.03 (0.48,2.18) | 1.78 (0.71,4.44) | 0.37 (0.08,1.61) | 1.15 (0.28,4.83) | 1.75 (0.26,11.91) | WSG+CWM |  |  |  |  |  |  |  |
| 0.29 (0.03,3.05) | 0.51 (0.05,5.60) | 0.11 (0.01,1.51) | 0.33 (0.02,4.62) | 0.50 (0.03,9.38) | 0.29 (0.03,2.99) | JWC+CWM |  |  |  |  |  |  |
| 0.91 (0.22,3.76) | 1.59 (0.35,7.15) | 0.33 (0.05,2.20) | 1.03 (0.16,6.65) | 1.56 (0.16,14.96) | 0.89 (0.21,3.70) | 3.12 (0.22,43.60) | WCAC+CWM |  |  |  |  |  |
| 0.75 (0.21,2.71) | 1.30 (0.33,5.19) | 0.27 (0.04,1.64) | 0.84 (0.14,4.95) | 1.28 (0.14,11.34) | 0.73 (0.20,2.66) | 2.56 (0.20,33.43) | 0.82 (0.14,4.78) | BWC+CWM |  |  |  |  |
| 0.76 (0.21,2.70) | 1.31 (0.33,5.18) | 0.27 (0.05,1.64) | 0.85 (0.15,4.95) | 1.29 (0.15,11.36) | 0.74 (0.21,2.65) | 2.59 (0.20,33.53) | 0.83 (0.14,4.78) | 1.01 (0.19,5.25) | STOL+CWM |  |  |  |
| 0.82 (0.31,2.17) | 1.42 (0.47,4.29) | 0.29 (0.06,1.46) | 0.92 (0.19,4.38) | 1.39 (0.18,10.47) | 0.80 (0.30,2.14) | 2.79 (0.25,31.69) | 0.89 (0.19,4.23) | 1.09 (0.26,4.58) | 1.08 (0.26,4.48) | LNC+CWM |  |  |
| 1.16 (0.36,3.73) | 2.02 (0.57,7.23) | 0.42 (0.07,2.35) | 1.31 (0.24,7.06) | 1.99 (0.24,16.46) | 1.14 (0.35,3.68) | 3.98 (0.32,49.04) | 1.28 (0.24,6.83) | 1.55 (0.32,7.46) | 1.54 (0.32,7.30) | 1.43 (0.38,5.41) | LAC+CWM |  |
| 0.90 (0.53,1.52) | 1.56 (0.75,3.26) | 0.32 (0.08,1.28) | 1.01 (0.27,3.81) | 1.54 (0.24,9.68) | 0.88 (0.51,1.51) | 3.08 (0.31,30.26) | 0.99 (0.26,3.68) | 1.20 (0.37,3.88) | 1.19 (0.37,3.79) | 1.10 (0.48,2.53) | 0.77 (0.27,2.19) | CWM |

**Appendix 7 CINeMA Assessment**

We use the CINeMA framework to evidence certainty, assessing it for each network estimate based on the following criteria:

- **Within study bias:** We classified the overall risk of bias for each study as low, moderate, or high when none of the four assessed risk of bias items were rated as high risk. See Appendix 3 for the bias assessment. The risk of bias for pairwise comparison of each intervention is shown in **Figure S5.1.**

**Figure S5.1:** Risk of bias contribution by intervention group in **total effective rate**


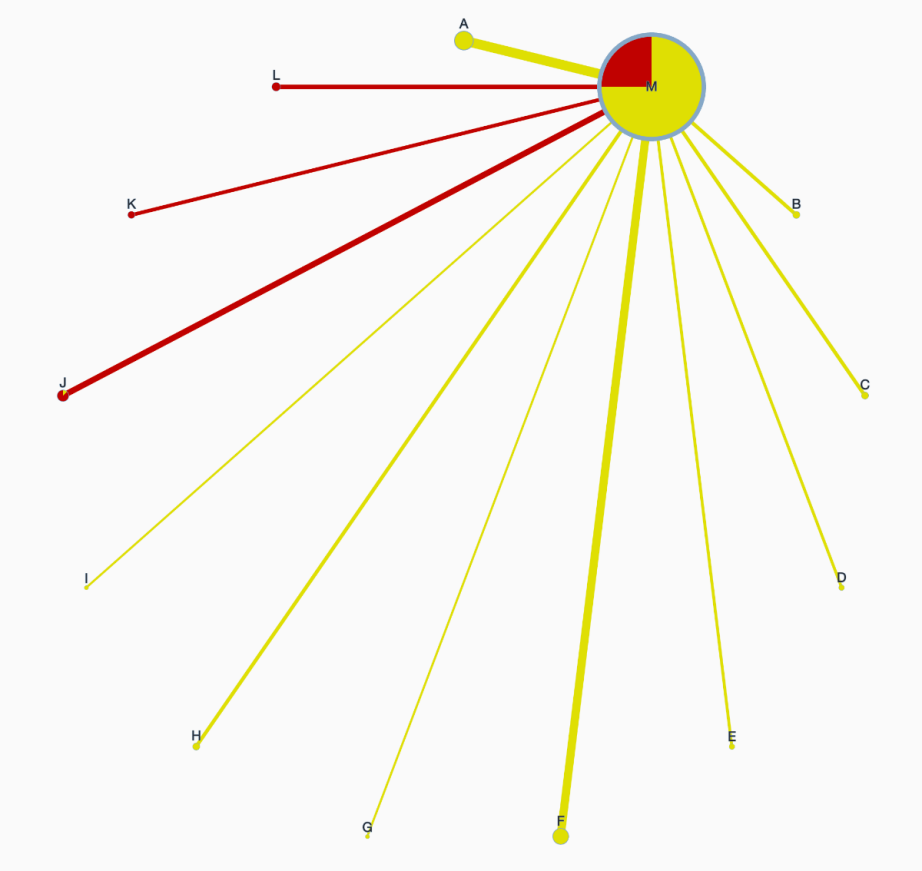


**Figure S5.2:** Overall risk of bias by treatment comparison in **Total effective rate**


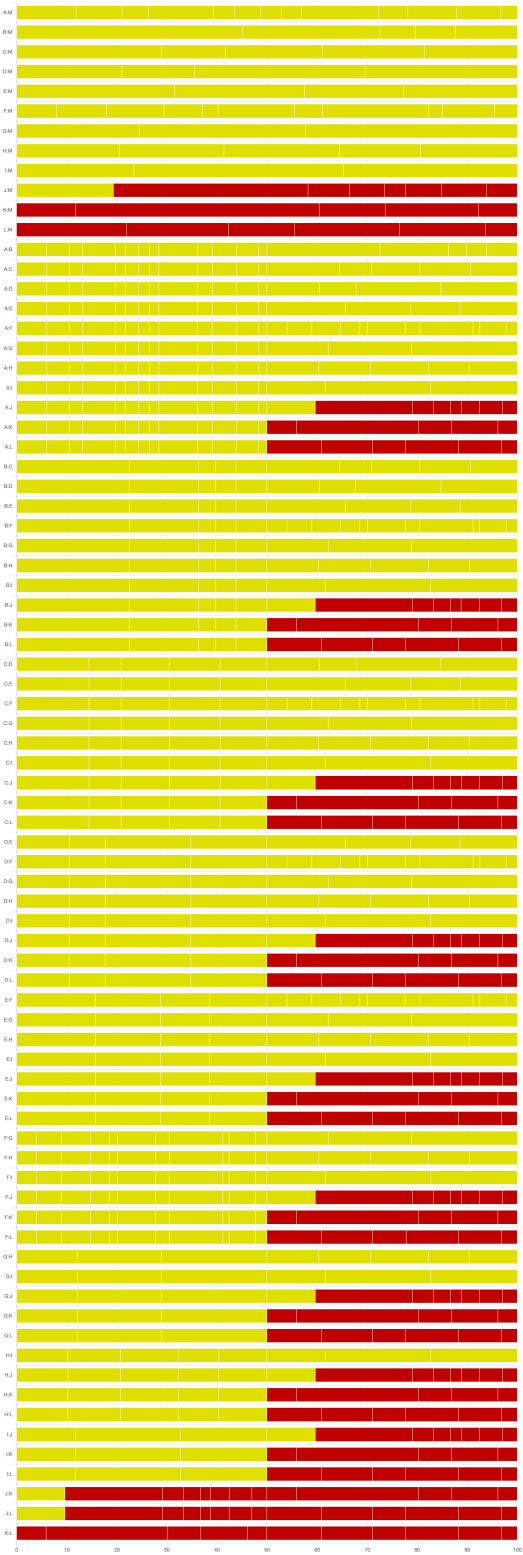


- **Reporting bias:** We assessed reporting bias within the CINeMA framework. Potential concerns were judged “suspected” when unpublished or gray literature was not included, when evidence relied mainly on a few early positive studies, or when selective reporting was likely. Otherwise, reporting bias was judged “undetected.” In addition, we examined comparison-adjusted funnel plots and conducted Egger’s regression tests in Stata to explore small-study effects.
- **Indirectness:** Transferability assumptions were assessed by comparing the mean age and disease duration at baseline across different intervention groups.

**Table S5.1:** Transitivity (Indirectness) Assessment

| Intervention | Baseline variable (Mean ± SD) | |
| --- | --- | --- |
|  | Age (year) | Disease duration (year) |
| ZKC+CWM | 44.59±9.60 | 4.60 ± 1.48 |
| WLC+CWM | 43.42±9.46 | 4.48 ± 1.32 |
| QWG+CWM | 47.98 ± 9.79 | 4.87 ± 0.36 |
| XPG+CWM | 41.02 ± 6.01 | 3.91 ± 0.47 |
| DLTG+CWM | 48.64 ± 2.57 | 3.36 ± 2.35 |
| WSG+CWM | 47.00 ± 6.32 | 3.55±0.27 |
| JWC+CWM | 44.76 ± 2.15 | 4.82±1.56 |
| WCAC+CWM | 43.67 ± 4.61 | 4.1±1.5 |
| BWC+CWM | 45.68 ± 3.31 | 3.51±0.24 |
| STOL+CWM | 46.28 ± 7.16 | 4.46±1.47 |
| LNC+CWM | 46.10±4.50 | 4.04±1.64 |
| LAC+CWM | 46.25±4. 67 | 4.59±1.24 |

- **Imprecision:** We use the CINeMA website to grade the accuracy of each comparison.
- **Heterogeneity:** We assessed the degree of worry by comparing clinical reasoning based on 95% confidence intervals (CIs) while applying the same clinical reasoning framework as for inaccuracy. In particular, we judged the consistency of our findings based on the confidence and prediction intervals associated with clinically important effect sizes. And we used the same thresholds of clinical significance as described above and followed the recommendations automatically provided by CINeMA (https://cinema.ispm.unibe.ch/).
- **Inconsistency:** Assessment of inconsistency was not feasible as the treatment network did not include any closed loops; consequently, node-splitting analyses could not be conducted.

**Table S5.2:** CINeMA Results of **total effective rate**

| Comparison | Within-study bias | Reporting bias | Indirectness | Imprecision | Heterogeneity | Incoherence | Confidence rating |
| --- | --- | --- | --- | --- | --- | --- | --- |
| A:M | Some concerns | Low risk | No concerns | No concerns | No concerns | Major concerns | Low |
| B:M | Some concerns | Low risk | No concerns | No concerns | No concerns | Major concerns | Low |
| C:M | Some concerns | Low risk | No concerns | No concerns | No concerns | Major concerns | Low |
| D:M | Some concerns | Low risk | No concerns | No concerns | Major concerns | Major concerns | Very low |
| E:M | Some concerns | Low risk | No concerns | No concerns | No concerns | Major concerns | Low |
| F:M | Some concerns | Low risk | No concerns | No concerns | No concerns | Major concerns | Low |
| G:M | Some concerns | Low risk | No concerns | No concerns | No concerns | Major concerns | Low |
| H:M | Some concerns | Low risk | No concerns | No concerns | No concerns | Major concerns | Low |
| I:M | Some concerns | Low risk | No concerns | No concerns | No concerns | Major concerns | Low |
| J:M | Major concerns | Low risk | No concerns | No concerns | No concerns | Major concerns | Very low |
| K:M | Major concerns | Low risk | No concerns | No concerns | No concerns | Major concerns | Very low |
| L:M | Major concerns | Low risk | No concerns | No concerns | No concerns | Major concerns | Very low |
| A:B | Some concerns | Low risk | No concerns | Major concerns | No concerns | Major concerns | Very low |
| A:C | Some concerns | Low risk | No concerns | Major concerns | No concerns | Major concerns | Very low |
| A:D | Some concerns | Low risk | No concerns | Major concerns | No concerns | Major concerns | Very low |
| A:E | Some concerns | Low risk | No concerns | Major concerns | No concerns | Major concerns | Very low |
| A:F | Some concerns | Low risk | No concerns | Major concerns | No concerns | Major concerns | Very low |
| A:G | Some concerns | Low risk | No concerns | No concerns | No concerns | Major concerns | Low |
| A:H | Some concerns | Low risk | No concerns | Major concerns | No concerns | Major concerns | Very low |
| A:I | Some concerns | Low risk | No concerns | Major concerns | No concerns | Major concerns | Very low |
| A:J | Some concerns | Low risk | No concerns | Major concerns | No concerns | Major concerns | Very low |
| A:K | Some concerns | Low risk | No concerns | Major concerns | No concerns | Major concerns | Very low |
| A:L | Major concerns | Low risk | No concerns | Major concerns | No concerns | Major concerns | Very low |
| B:C | Some concerns | Low risk | No concerns | Major concerns | No concerns | Major concerns | Very low |
| B:D | Some concerns | Low risk | No concerns | No concerns | No concerns | Major concerns | Very low |
| B:E | Some concerns | Low risk | No concerns | Major concerns | No concerns | Major concerns | Very low |
| B:F | Some concerns | Low risk | No concerns | Major concerns | No concerns | Major concerns | Very low |
| B:G | Some concerns | Low risk | No concerns | Major concerns | No concerns | Major concerns | Very low |
| B:H | Some concerns | Low risk | No concerns | Major concerns | No concerns | Major concerns | Very low |
| B:I | Some concerns | Low risk | No concerns | Major concerns | No concerns | Major concerns | Very low |
| B:J | Some concerns | Low risk | No concerns | Major concerns | No concerns | Major concerns | Very low |
| B:K | Some concerns | Low risk | No concerns | Major concerns | No concerns | Major concerns | Very low |
| B:L | Major concerns | Low risk | No concerns | Major concerns | No concerns | Major concerns | Very low |
| C:D | Some concerns | Low risk | No concerns | No concerns | Major concerns | Major concerns | Very low |
| C:E | Some concerns | Low risk | No concerns | Major concerns | No concerns | Major concerns | Very low |
| C:F | Some concerns | Low risk | No concerns | Major concerns | No concerns | Major concerns | Very low |
| C:G | Some concerns | Low risk | No concerns | Major concerns | No concerns | Major concerns | Very low |
| C:H | Some concerns | Low risk | No concerns | Major concerns | No concerns | Major concerns | Very low |
| C:I | Some concerns | Low risk | No concerns | Major concerns | No concerns | Major concerns | Very low |
| C:J | Some concerns | Low risk | No concerns | Major concerns | No concerns | Major concerns | Very low |
| C:K | Some concerns | Low risk | No concerns | Major concerns | No concerns | Major concerns | Very low |
| C:L | Some concerns | Low risk | No concerns | Major concerns | No concerns | Major concerns | Very low |
| D:E | Some concerns | Low risk | No concerns | Major concerns | No concerns | Major concerns | Very low |
| D:F | Some concerns | Low risk | No concerns | Major concerns | No concerns | Major concerns | Very low |
| D:G | Some concerns | Low risk | No concerns | No concerns | No concerns | Major concerns | Low |
| D:H | Some concerns | Low risk | No concerns | Major concerns | No concerns | Major concerns | Very low |
| D:I | Some concerns | Low risk | No concerns | Major concerns | No concerns | Major concerns | Very low |
| D:J | Some concerns | Low risk | No concerns | Major concerns | No concerns | Major concerns | Very low |
| D:K | Some concerns | Low risk | No concerns | Major concerns | No concerns | Major concerns | Very low |
| D:L | Some concerns | Low risk | No concerns | Major concerns | No concerns | Major concerns | Very low |
| E:F | Some concerns | Low risk | No concerns | Major concerns | No concerns | Major concerns | Very low |
| E:G | Some concerns | Low risk | No concerns | No concerns | Major concerns | Major concerns | Very low |
| E:H | Some concerns | Low risk | No concerns | Major concerns | No concerns | Major concerns | Very low |
| E:I | Some concerns | Low risk | No concerns | Major concerns | No concerns | Major concerns | Very low |
| E:J | Some concerns | Low risk | No concerns | Major concerns | No concerns | Major concerns | Very low |
| E:K | Some concerns | Low risk | No concerns | Major concerns | No concerns | Major concerns | Very low |
| E:L | Some concerns | Low risk | No concerns | Major concerns | No concerns | Major concerns | Very low |
| F:G | Some concerns | Low risk | No concerns | No concerns | No concerns | Major concerns | Very low |
| F:H | Some concerns | Low risk | No concerns | Major concerns | No concerns | Major concerns | Very low |
| F:I | Some concerns | Low risk | No concerns | Major concerns | No concerns | Major concerns | Very low |
| F:J | Some concerns | Low risk | No concerns | Major concerns | No concerns | Major concerns | Very low |
| F:K | Some concerns | Low risk | No concerns | Major concerns | No concerns | Major concerns | Very low |
| F:L | Major concerns | Low risk | No concerns | Major concerns | No concerns | Major concerns | Very low |
| G:H | Some concerns | Low risk | No concerns | No concerns | No concerns | Major concerns | Very low |
| G:I | Some concerns | Low risk | No concerns | No concerns | No concerns | Major concerns | Very low |
| G:J | Some concerns | Low risk | No concerns | No concerns | No concerns | Major concerns | Very low |
| G:K | Some concerns | Low risk | No concerns | No concerns | No concerns | Major concerns | Very low |
| G:L | Some concerns | Low risk | No concerns | No concerns | No concerns | Major concerns | Very low |
| H:I | Some concerns | Low risk | No concerns | Major concerns | No concerns | Major concerns | Very low |
| H:J | Some concerns | Low risk | No concerns | Major concerns | No concerns | Major concerns | Very low |
| H:K | Some concerns | Low risk | No concerns | Major concerns | No concerns | Major concerns | Very low |
| H:L | Some concerns | Low risk | No concerns | Major concerns | No concerns | Major concerns | Very low |
| I:J | Some concerns | Low risk | No concerns | Major concerns | No concerns | Major concerns | Very low |
| I:K | Some concerns | Low risk | No concerns | Major concerns | No concerns | Major concerns | Very low |
| I:L | Some concerns | Low risk | No concerns | Major concerns | No concerns | Major concerns | Very low |
| J:K | Major concerns | Low risk | No concerns | Major concerns | No concerns | Major concerns | Very low |
| J:L | Major concerns | Low risk | No concerns | Major concerns | No concerns | Major concerns | Very low |
| K:L | Major concerns | Low risk | No concerns | Major concerns | No concerns | Major concerns | Very low |

Note: A = ZKC + CWM; B = WLC + CWM; C = QWG + CWM; D = XPG + CWM; E = DLTG + CWM; F = WSG + CWM; G = JWC + CWM; H = WCAC + CWM; I = BWC + CWM; J = STOL + CWM; K = LNC + CWM; L = LAC + CWM; M = CWM

**Appendix 8. Funnel plots of publication bias**

Figure S1. Funnel plot for total effective rate.


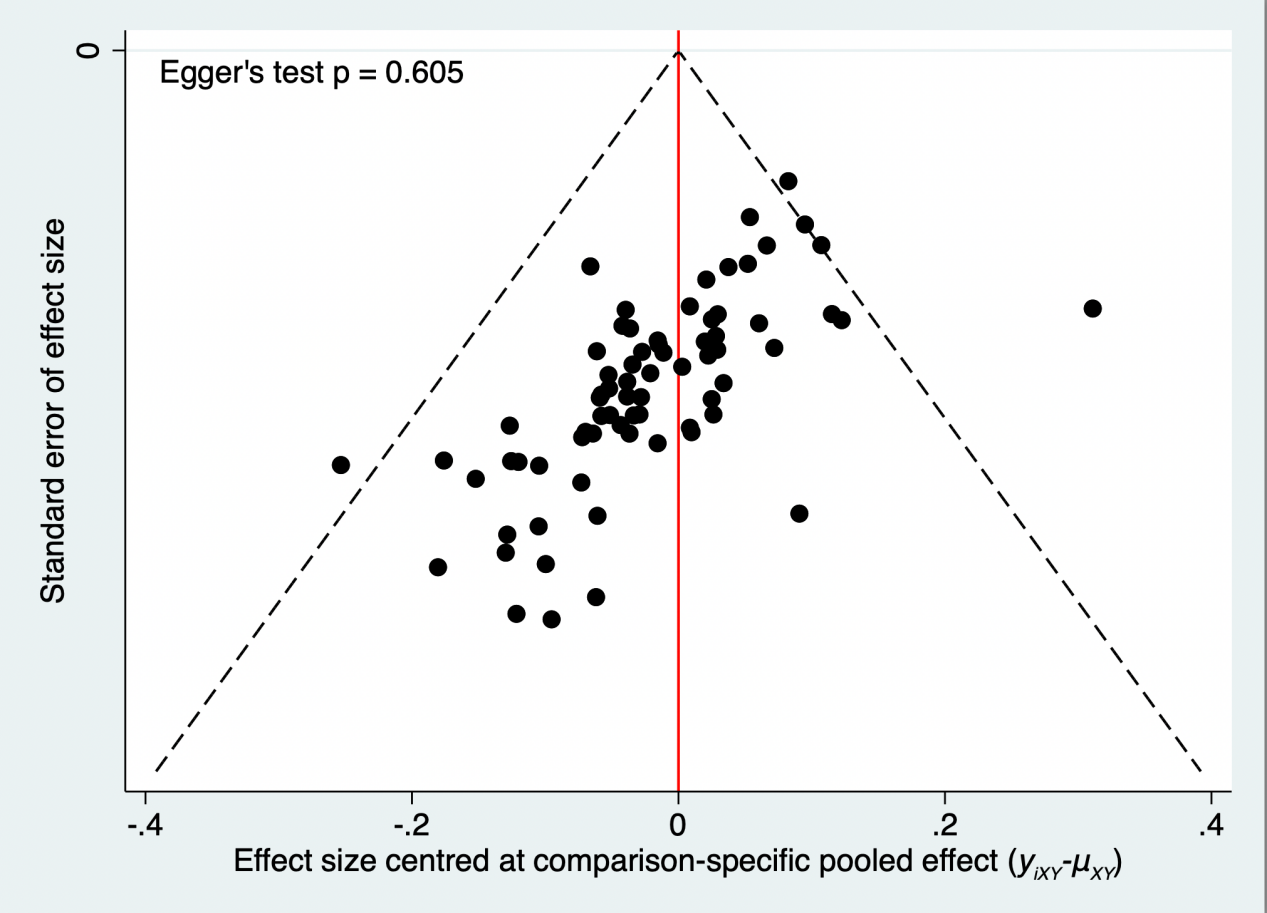


Figure S2. Funnel plot for motilin (MTL) levels.


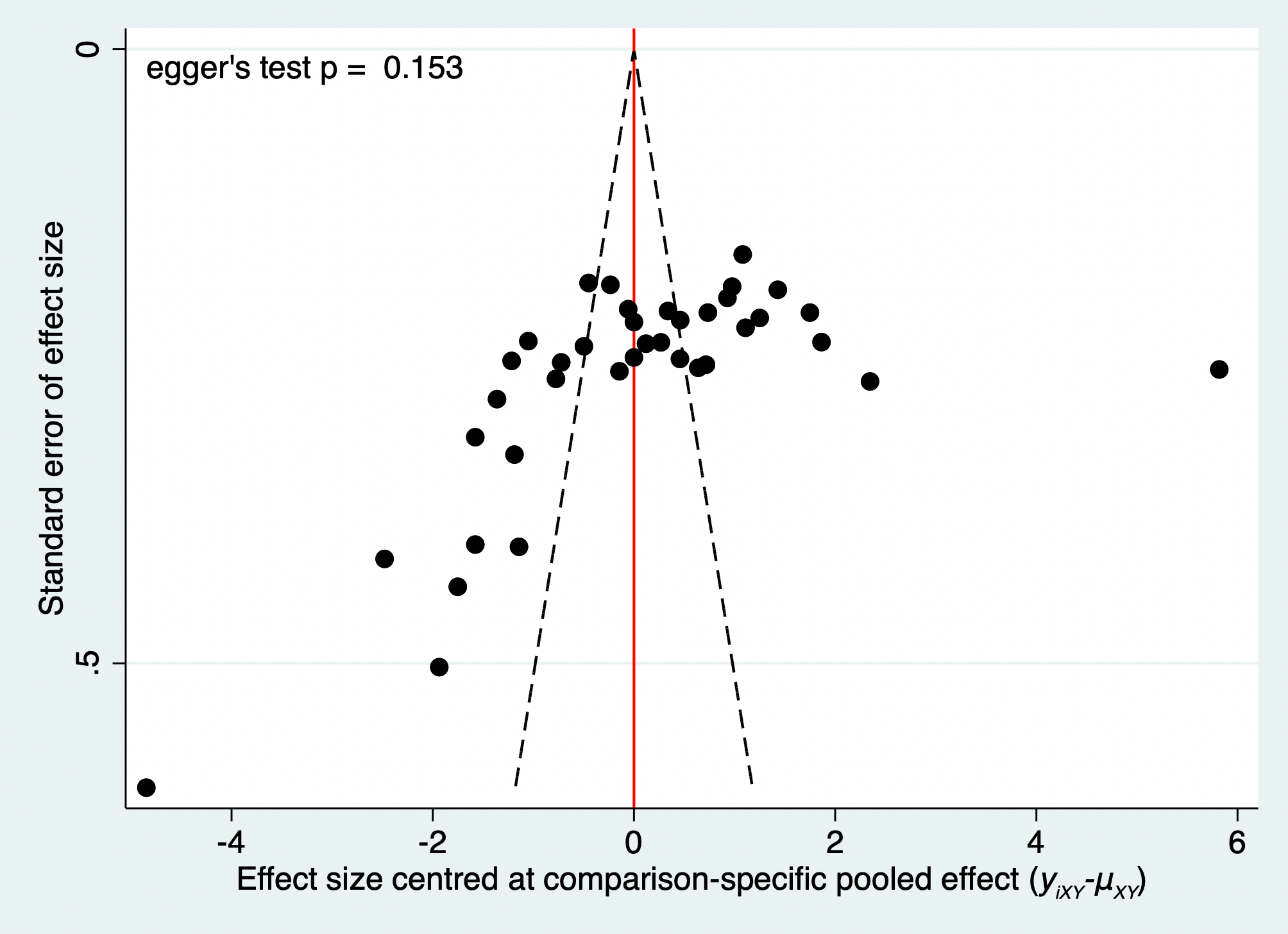


Figure S3. Funnel plot for gastrin (GAS) levels


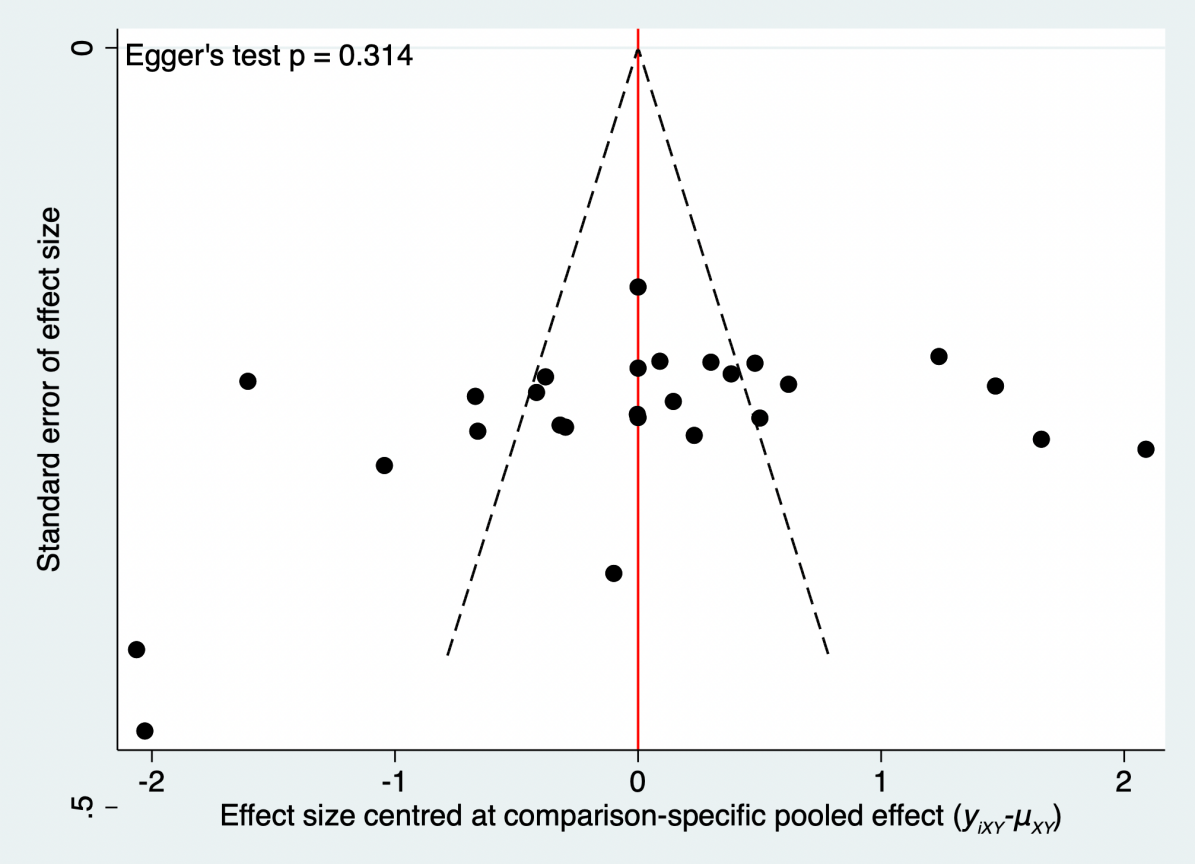


Figure S4. Funnel plot for adverse events


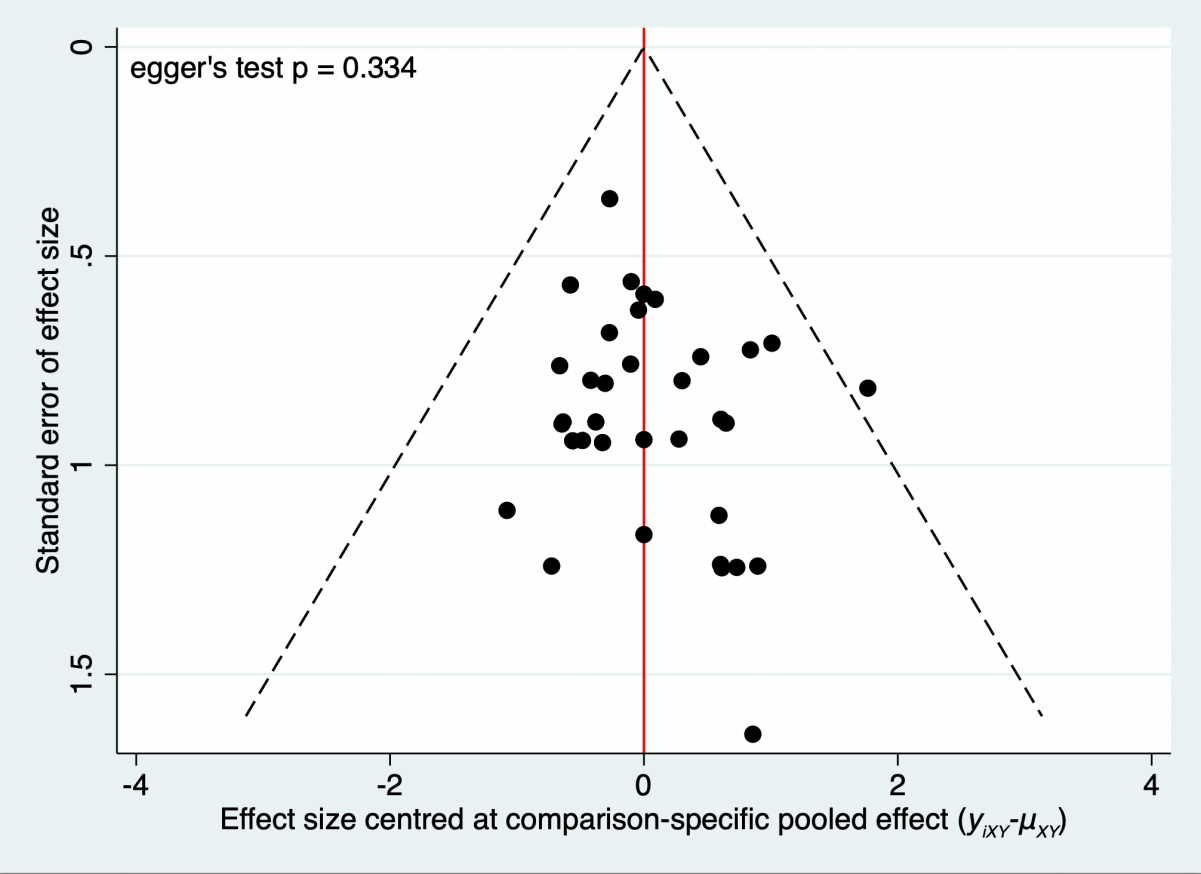


**Appendix 9. Sensitivity Analyses**

| Intervention | Total effective rate | | | MTL | | | GAS | | | Adverse event | | |
| --- | --- | --- | --- | --- | --- | --- | --- | --- | --- | --- | --- | --- |
|  | Original | Excluding small-sample studies | Excluding high-risk studies | Original | Excluding small-sample studies | Excluding high-risk studies | Original | Excluding small-sample studies | Excluding high-risk studies | Original | Excluding small-sample studies | Excluding high-risk studies |
| ZKC+CWM | 1.20 (1.13,1.26) | 1.19 (1.12–1.26) | 1.21 (1.14–1.28) | 2.62 (1.41,3.84) | 2.55 (1.35–3.76) | 2.68 (1.45–3.92) | 0.60 (-0.33,1.53) | 0.58 (−0.35–1.50) | 0.62 (−0.30–1.55) | 0.90 (0.53,1.52) | 0.92 (0.55–1.54) | 0.89 (0.52–1.50) |
| WLC+CWM | 1.29 (1.18,1.42) | 1.28 (1.17–1.41) | 1.30 (1.19–1.44) | 2.85 (0.15,5.56) | 2.78 (0.10–5.47) | 2.89 (0.20–5.60) | 1.37 (-0.88,3.61) | 1.32 (−0.92–3.55) | 1.40 (−0.85–3.65) | 1.56 (0.75,3.26) | 1.60 (0.78–3.30) | 1.58 (0.76–3.28) |
| QWG+CWM | 1.26 (1.14,1.38) | 1.25 (1.13–1.37) | 1.27 (1.15–1.39) | 0.35 (-2.34,3.05) | 0.40 (−2.20–3.00) | 0.32 (−2.40–3.08) |  |  |  | 0.32 (0.08,1.28) | 0.35 (0.09–1.30) | 0.33 (0.08–1.27) |
| XPG+CWM | 1.11 (1.01,1.22) | 1.12 (1.02–1.23) | 1.10 (1.00–1.21) | 0.90 (-0.81,2.60) | 0.88 (−0.85–2.62) | 0.92 (−0.75–2.65) | 1.25 (0.24,2.27) | 1.28 (0.22–2.30) | 1.22 (0.20–2.25) | 1.01 (0.27,3.81) | 1.05 (0.28–3.90) | 1.00 (0.26–3.80) |
| DLTG+CWM | 1.24 (1.12,1.37) | 1.23 (1.11–1.36) | 1.25 (1.13–1.38) | 1.90 (-0.31,4.11) | 1.85 (−0.35–4.05) | 1.95 (−0.28–4.18) | 2.00 (0.68,3.33) | 2.05 (0.70–3.38) | 1.95 (0.65–3.28) | 1.54 (0.24,9.68) | 1.50 (0.23–9.60) | 1.52 (0.25–9.65) |
| WSG+CWM | 1.20 (1.13,1.27) | 1.19 (1.12–1.26) | 1.21 (1.14–1.28) | 1.47 (-0.73,3.66) | 1.42 (−0.78–3.60) | 1.50 (−0.68–3.72) | 1.57 (0.26,2.88) | 1.60 (0.28–2.92) | 1.55 (0.25–2.85) | 0.88 (0.51,1.51) | 0.86 (0.50–1.49) | 0.90 (0.52–1.53) |
| JWC+CWM | 1.46 (1.28,1.67) | 1.45 (1.27–1.66) | 1.44 (1.26–1.65) |  |  |  |  |  |  | 3.08 (0.31,30.26) | 3.02 (0.30–30.10) | 3.10 (0.32–30.40) |
| WCAC+CWM | 1.21 (1.11,1.32) | 1.22 (1.12–1.33) | 1.20 (1.10–1.31) | 1.45 (-0.75,3.65) | 1.50 (−0.70–3.68) | 1.42 (−0.80–3.62) | 1.91 (-0.36,4.19) | 1.88 (−0.38–4.15) | 1.92 (−0.34–4.22) | 0.99 (0.26,3.68) | 1.02 (0.27–3.75) | 0.97 (0.25–3.66) |
| BWC+CWM | 1.20 (1.08,1.34) | 1.21 (1.09–1.34) | 1.19 (1.07–1.33) | 0.91 (-2.89,4.72) | 0.95 (−2.80–4.70) | 0.89 (−2.95–4.65) | 0.03 (-2.23,2.30) | 0.05 (−2.20–2.32) | 0.01 (−2.25–2.27) | 1.20 (0.37,3.88) | 1.18 (0.36–3.85) | 1.22 (0.38–3.90) |
| STOL+CWM | 1.18 (1.11,1.26) | 1.17 (1.10–1.25) | 1.18 (1.10–1.26) | 2.07 (0.17,3.98) | 2.02 (0.15–3.90) | 2.15 (0.20–4.05) | 1.94 (0.63,3.25) | 1.97 (0.65–3.28) | 1.90 (0.60–3.20) | 1.19 (0.37,3.79) | 1.15 (0.35–3.72) | 1.17 (0.36–3.77) |
| LNC+CWM | 1.20 (1.11,1.31) | 1.19 (1.10–1.30) | 1.21 (1.12–1.32) | 1.80 (-0.10,3.71) | 1.75 (−0.15–3.65) | 1.82 (−0.12–3.74) | 2.75 (1.59,3.91) | 2.70 (1.55–3.85) | 2.80 (1.62–3.98) | 1.10 (0.48,2.53) | 1.12 (0.50–2.55) | 1.08 (0.47–2.50) |
| LAC+CWM | 1.21 (1.12,1.32) | 1.22 (1.13–1.33) | 1.20 (1.11–1.31) | 3.21 (-0.60,7.02) | 3.10 (−0.65–6.95) | 3.25 (−0.55–7.10) |  |  |  | 0.77 (0.27,2.19) | 0.80 (0.28–2.22) | 0.75 (0.26–2.17) |

**Appendix 10: The meta-regression of the factors that may lead to differences to the outcome indicators**

| Factor | Total effective rate | | UPDRS | | Webster | | HAMD | |
| --- | --- | --- | --- | --- | --- | --- | --- | --- |
|  | B | 95% CI | B | 95% CI | B | 95% CI | B | 95% CI |
| Age | -0.16 | -0.86, 1.06 | 0.13 | -0.35, 1.52 | -0.13 | -2.03, 1.79 | -0.31 | -2.85, 0.92 |
| Disease duration | -0.27 | -0.15, 0.50 | 0.31 | -0.96, 1.25 | -0.4 | -1.51, 0.94 | -0.24 | -2.50, 1.63 |
| Treatment duration (>4 vs ≤4 weeks) | 0.18 | -0.93, 1.00 | 0.24 | -0.83, 1.27 | -0.25 | -1.07, 0.71 | 0.15 | -1.35, 1.69 |
